# *SMALL GRAIN 5* encodes a heat shock transcription factor controlling grain size and plant architecture in rice

**DOI:** 10.3389/fpls.2026.1783348

**Published:** 2026-04-01

**Authors:** Chenjie Wang, Nannan Han, Zhao Li, Chen Zhou, Guansong Li, Yongqing Pan, Baolan Zhang, Ke Huang, Yunhai Li, Yingjie Li

**Affiliations:** 1 School of Breeding and Multiplication (Sanya Institute of Breeding and Multiplication), Hainan University, Sanya, China; 2 Key Laboratory of Seed Innovation, Institute of Genetics and Developmental Biology, Chinese Academy of Sciences, Beijing, China; 3 University of Chinese Academy of Sciences, Beijing, China; 4 State Key Laboratory of Crop Stress Adaptation and Improvement, School of Life Sciences, Henan University, Kaifeng, China; 5 Hainan Seed Industry Laboratory, Sanya, China

**Keywords:** grain size, heat shock transcription factor, plant architecture, rice, *SMG5*, transcriptional regulation

## Abstract

Grain size is a critical determinant of rice yield. Despite the identification of several genes involved in grain size regulation, the underlying molecular mechanisms remain incompletely understood. A comprehensive understanding of the genetic and molecular mechanisms that regulate seed size is crucial for improving crop yield. Here, we report the characterization of *SMALL GRAIN 5* (*SMG5*), which encodes a heat shock transcription factor (HSF) and serves as a key regulator of both grain size and plant architecture in rice. The *smg5* mutant exhibits pleiotropic phenotypes, including significantly reduced grain size, as well as decreased plant height, leaf width, tiller number, and grain number per panicle. *SMG5* regulates grain size through both cell expansion and cell proliferation. It is expressed in developing panicles, and the GFP-SMG5 fusion protein is localized in the nucleus. Integrated RNA-seq and DAP-seq analyses reveal that SMG5 binds to the promoter region of *DGS1* and directly modulates its expression. Notably, overexpression of *SMG5* resulted in larger grains. These findings establish SMG5 as a key HSF that modulates grain size and plant architecture, providing insights into the regulatory network controlling grain development in rice.

## Introduction

Rice is a staple food for over half of the world’s population. Grain yield, a complex trait, is determined by three key components: panicle number, grain number per panicle, and grain weight (Xing and Zhang, 2010). Grain size, defined by its length, width, and thickness, is a critical determinant of both grain weight and appearance, making it a primary target for yield enhancement (Zuo and Li, 2014).

The final size of the rice grain is physically constrained by the spikelet hull, whose growth is coordinately regulated by cell proliferation and cell expansion (Li and Li, 2016; Li et al., 2019). Several genes have been identified that regulate grain size through the modulation of cell division. For instance, *GW2*, which encodes an E3 ubiquitin ligase, functions as a negative regulator of grain width. A loss-of-function of *GW2* increases the number of cells in the spikelet hulls, resulting in wider grains and increased grain weight (Song et al., 2007). Another quantitative trait locus (QTL) *GW5*/*GSE5*, encodes a calmodulin-binding protein. A natural deletion in its promoter region is associated with reduced expression level of *GSE5* and increased grain width (Duan et al., 2017). GW5/GSE5 physically interacts with the kinase GSK2, inhibiting its activity and modulating the expression of brassinosteroid (BR)-responsive genes to influence growth (Liu et al., 2017). GS3, a major quantitative trait locus (QTL) for grain length, negatively regulates grain size by limiting cell division in the spikelet hull (Mao et al., 2010). In contrast, *GS5* and *OsSPL16*/*GW8* promote grain size by increasing cell number (Li et al., 2011; Wang et al., 2012). A mitogen-activated protein kinase (MAPK) module consisting of OsMKKK10, OsMKK4, and OsMAPK6 plays a central role in grain size by promoting cell proliferation (Duan et al., 2014; Guo et al., 2018; Liu et al., 2015; Xu et al., 2018a, b). Cell expansion also plays a crucial role in determining grain size. The transcription factor *SPL13*/*GWL7* promotes grain length by enhancing cell elongation in the spikelet hull (Si et al., 2016). Similarly, OsGRF4/GS2, primarily enhances grain size by stimulating cell expansion (Duan et al., 2015; Hu et al., 2015; Li et al., 2016; Sun et al., 2016). *WTG1*/*OsOTUB1*, which encodes a deubiquitinating enzyme, influences cell expansion. The *wtg1–1* mutant exhibits wide, thick, and short grains with increased grain weight, and WTG1/OsOTUB1 interacts with IPA1 to regulate plant architecture (Huang et al., 2017; Wang et al., 2017). Furthermore, *LARGE1*, which encodes a MEI2-like RNA-binding protein, serves as a negative regulator of grain size. LARGE1 is phosphorylated by GSK2 and participates in BR-mediated grain development (Lyu et al., 2020).

Heat shock transcription factors (HSFs) are well-known master regulators of heat stress response, but emerging evidence suggests their involvement in various developmental processes. For example, OsHsfA4a confers cadmium tolerance by activating metallothionein gene expression (Shim et al., 2009). OsHsfB2b, OsHsfA7, and OsHsfC1b contribute to salt and drought tolerance (Liu et al., 2013; Schmidt et al., 2012; Xiang et al., 2013). Notably, OsHsfC1b has been shown to increase seed weight, size, and vigor, suggesting a potential role in yield formation (Achary et al., 2025). However, despite these advances, the roles of HSFs in regulating key agronomic traits, particularly grain size and yield under normal growth conditions, remain largely unexplored.

In this study, we isolated a recessive small grain mutant, *small grain 5* (*smg5*), which exhibits pleiotropic defects in plant architecture and grain development. We demonstrate that *SMG5* encodes a heat shock transcription factor and is an allele of *OsHsfA4d*/*SPL7*. Our findings show that SMG5 localizes to the nucleus and functions as a transcriptional regulator, directly binding to and activating the expression of *DGS1*, thereby coordinating cell proliferation and expansion to control both grain size and plant architecture.

## Materials and methods

### Plant materials and growth conditions

Seeds of the japonica rice variety Zhonghua11 (ZH11) were mutagenized with ethyl methanesulfonate (EMS), and the *small grain 5* (*smg5*) mutant was identified from the M_2_ population. Rice plants were cultivated in paddy fields with a planting density of 20 cm × 20 cm in Lingshui (Hainan, China) and Beijing during natural growing conditions.

### Morphological and cellular analysis

Plants and grains were photographed and measured at maturity. Grains from main panicles were scanned using a MICROTEK Scan Marker i560 (MICROTEK, Shanghai, China). Grain length and width were measured using the WSEEN Rice Test System (WSeen, Zhejiang, China). Grain thickness was measured using a digital caliper (JIANYE TOOLS, Zhejinag, China). Grain weight was determined based on three biological replicates, each consisting of 100 dried grains.

Mature grains were observed using scanning electron microscopy (SEM) after gold spraying treatment. Outer epidermal cell size in the central region of lemmas was measured using ImageJ software. Cell numbers were counted along the longest part for grain-length direction and the widest part for grain-width direction.

### Identification of SMG5

The *smg5* mutant was crossed with ZH11 to generate an F_2_ population. Pooled DNA from 50 F_2_ individuals exhibiting the small-grain phenotype was subjected to whole-genome resequencing using the Illumina NextSeq 500 platform. Candidate causal mutations were identified using the MutMap method as previously described (Abe et al., 2012; Fang et al., 2016; Huang et al., 2017). A single SNP (SNP2) with an SNP/INDEL-index = 1 was identified in the exon of *LOC_Os05g45410*.

### Constructs and plant transformation

For genetic complementation, the primers C99-SMG5-F and C99-SMG5-R were used to amplify the genomic sequence of *SMG5*, including 2000 bp of the 5’ flanking sequence, the full coding region, and 1000 bp of the 3’ flanking sequence. The genomic sequence of *SMG5* was cloned into the pMDC99 vector using the ClonExpress Ultra One Step Cloning Kit (Vazyme, C115-01) to generate the *gSMG5* construct. For subcellular localization and ChIP-qPCR, the coding sequence (CDS) of the SMG5 was simplified using primers GFP-SMG5-F and GFP-SMG5-R and inserted into the pMDC43 vector to generate the pro35S:GFP-SMG5 construct. All the constructs were introduced into *Agrobacterium tumefaciens* strain *GV3101* and transformed into *smg5* or ZH11 plants.

### Subcellular localization of SMG5

Coleoptiles of *pro35S*:*GFP*-*SMG5* transgenic plants were used for subcellular localization analysis. GFP fluorescence was observed using a Zeiss LSM 980 confocal microscope. Nuclei were stained with DAPI (1 μg/mL).

### RNA extraction, qRT-PCR, and RNA-seq analysis

Total RNA was extracted from young panicles using an RNA extraction kit (ZOMANBIO, ZP405K-2). First-strand cDNA was synthesized using the cDNA Synthesis Kit (Vazyme, R211). Quantitative real-time PCR was performed using SYBR qPCR Mix (Genstar, A301-10) on a LightCycler 480 (Roche, Switzerland). Rice *ACTIN1* was used as an internal control. Primers are listed in **Supplementary Table 2**.

For RNA-seq analysis, total RNA was isolated from young panicles of ZH11 and *smg5*, with three biological replicates each. The libraries were constructed, and sequencing was performed by BerryGenomics Corporation on the Illumina NovaSeq 6000 platform using 150-bp double-end sequencing. Clean reads were aligned to the Nipponbare reference genome (MSU7.0) using TopHat and Bowtie 2 software.

### DAP-seq analyses

DNA affinity purification sequencing (DAP-seq) was performed as previously described (Bartlett et al., 2017; Huang et al., 2024). To express the SMG5 protein *in vitro*, the CDS of *SMG5* was amplified using primers GST-SMG5-F and GST-SMG5-R and cloned into pGEX4T-1. The GST-SMG5 fusion protein was expressed in *E. coli* BL21 (DE3) and purified using Glutathione Sepharose 4B beads (Cytiva, 17075601). Purified GST-SMG5 (2 μg) was incubated with Glutathione Sepharose 4B beads in PBS buffer for 1 h at room temperature. The beads incubated with a genomic DNA library. After extensive washing, bound DNA fragments were eluted, amplified, and sequenced on the Illumina NovaSeq 6000 platform. Clean reads were aligned to the Nipponbare reference genome (MSU7.0) using Bowtie 2 software. Peak calling was performed using MACS2 software, and motif enrichment analysis was conducted using MEME-ChIP.

### Electrophoretic mobility shift assay

The GST-SMG5 fusion protein was expressed in *E. coli* BL21 and purified. Biotin-labeled DNA probes containing the TTCTTGAA motif and corresponding mutated probes were synthesized for binding assays. The Light Shift Chemiluminescent EMSA kit (Thermo Fisher Scientific, 20148) was used to perform electrophoretic mobility shift assay according to the manufacturer’s instructions. Probe sequences were listed in **Supplementary Table 2**.

### Chromatin immunoprecipitation and quantitative real-time PCR analysis

Chromatin immunoprecipitation (ChIP) assay was performed with minor modifications to previously described protocols. Young panicles from *pro35S*:*GFP*-*SMG5* transgenic plants were collected for ChIP assay. The samples were cross-linked with 1% formaldehyde for 15 min and quenched with 0.125 M glycine. Chromatin was sonicated to 200–500 bp fragments using a Bioruptor Pico (Diagenode, Belgium). The anti-GFP antibodies (Abcam, AB290) and protein A+G beads (Millipore, 16-663) were used for Immunoprecipitations. The precipitated DNA was recovered by using the QIAquick PCR Purification Kit (QIAGEN, 28106) and analyzed by qRT-PCR with primers targeting promoter regions of candidate genes. Primer sequences were listed in **Supplementary Table 2**.

### Dual-luciferase assay in rice protoplasts

The coding sequence of *OsSMG5* was cloned into the pMDC43 vector and used as the effector. The emptypMDC43 vector serving as the negative control. The 3,085 bp promoter region of *OsDGS1* was amplified and cloned into the pGreenII 0800-Luc reporter vector, which contains both the firefly luciferase (LUC) reporter gene and the Renilla luciferase (REN) internal control. The primers used for amplification are listed in **Supplementary Table 2**. Transcriptional activation assays were performed in protoplasts isolated from leaves of 10-day-old ZH11 seedlings, following previously described protocols with minor modifications (Hao et al., 2010; Zhang et al., 2011). After transformation, protoplasts were incubated in the dark at room temperature for 10 h, collected by centrifugation, and lysed. Firefly and Renilla luciferase activities were measured using the Dual-Luciferase Reporter Assay System (Promega, E1960, Madison, WI, USA) according to the manufacturer’s instructions. Transcriptional activation activity was calculated as the ratio of LUC to REN. Three biological replicates were performed for each assay.

## Results

### The *smg5* mutant produces small grains

**Figure 1:**
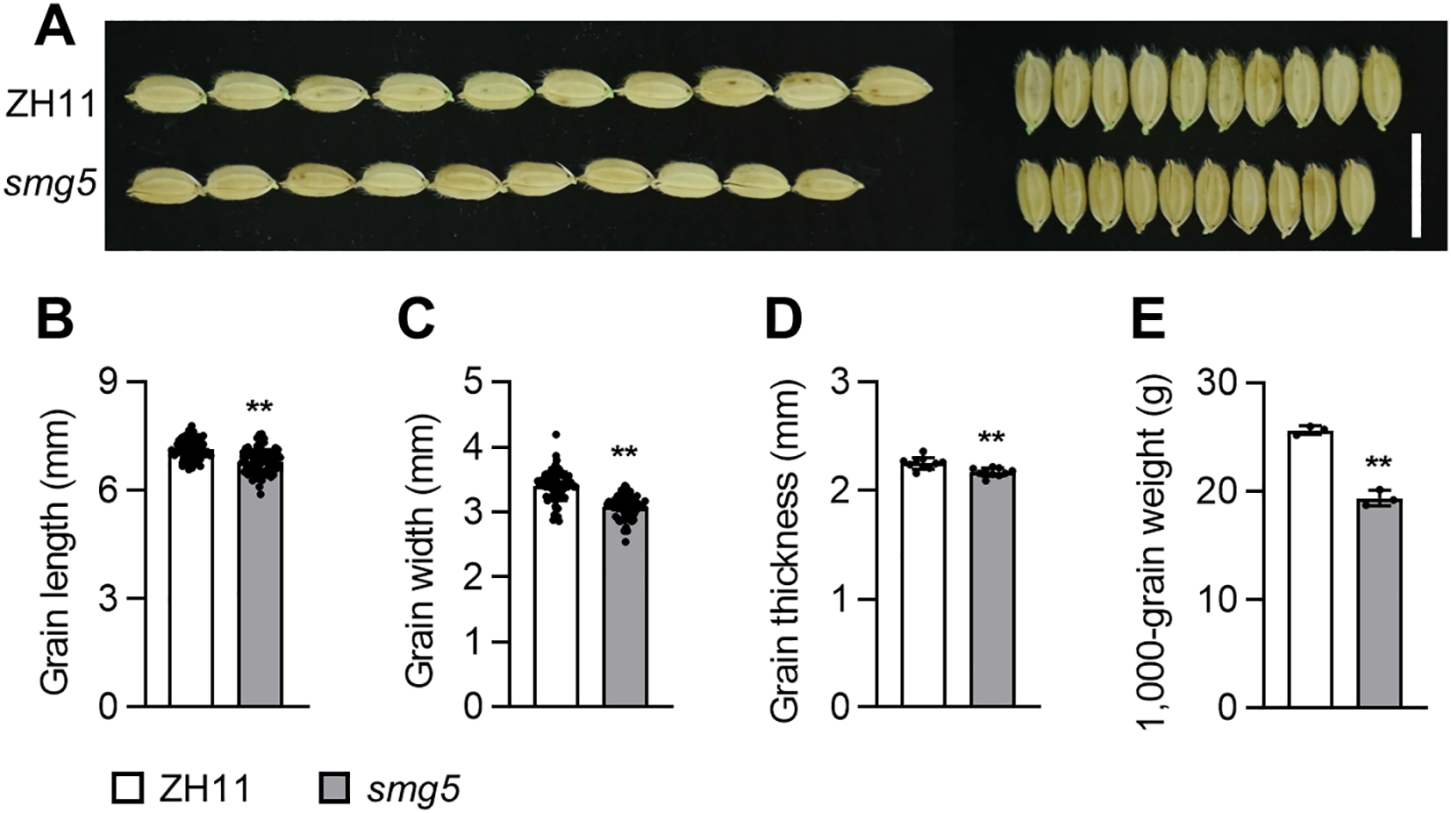
*SMG5* regulates grain size and weight. **(A)** Mature grains of the ZH11 and *smg5* mutant. **(B-E)**, The grain length **(B)**, grain width **(C)**, grain thickness **(D)**, and 1,000 grain weight **(E)** of ZH11 and *smg5*. n = 89 **(B, C)**, n = 10 **(D)**, and n =3 **(E)**. Values in **(B-E)** are given as mean ± SD. **P < 0.01 compared with ZH11 by Student’s *t*-test. Bars: 1 cm **(A)**.

To identify novel regulators of grain size, we screened an ethyl methanesulfonate (EMS)-mutagenized population of the Japonica cultivar Zhonghua11 (ZH11) and isolated a mutant with smaller grains, which we named *smg5* (**Figure 1A**). Compared to ZH11, the length of *smg5* grains was significantly reduced by 4.65% (**Figure 1B**). Similarly, the width of *smg5* grains was reduced by 9.42% compared with that of ZH11 grains (**Figure 1C**). The *smg5* grains were also significantly thinner and lighter than those of ZH11 (**Figures 1D, E**). The thickness and 1000-grain weight of *smg5* grains were significantly reduced by 3.69% and 24.38%, respectively, compared to ZH11 grains (**Figures 1D, E**). These results indicate that *SMG5* is a crucial regulator of grain size and weight in rice.

**Figure 2:**
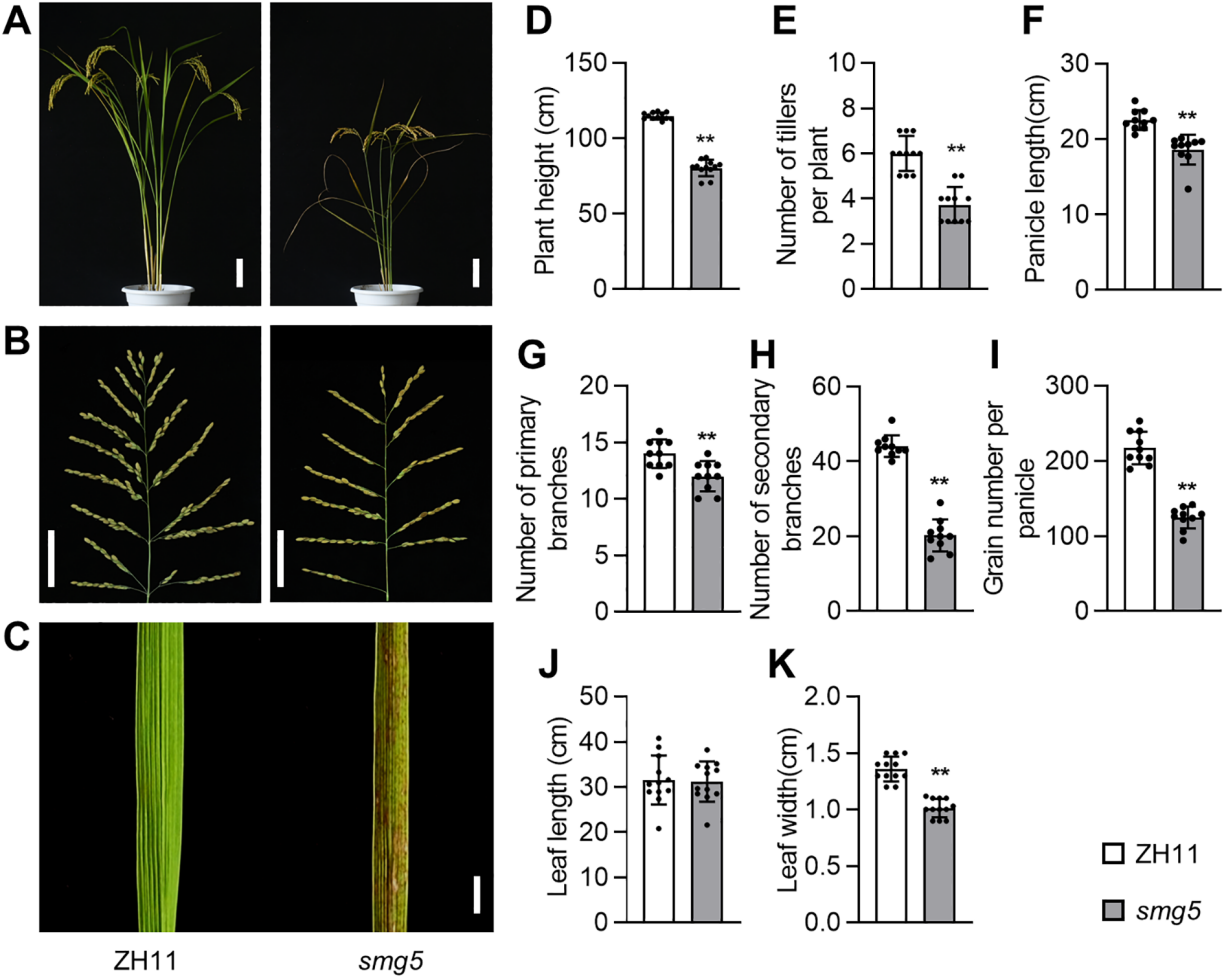
*SMG5* regulates plant architecture, panicle development and leaf morphology. **(A-C)** Mature plants **(A)**, panicles **(B)** and leaves **(C)** of the ZH11 and *smg5* mutant. **(D-E)**, The plant height **(D)** and tilling number **(E)** of ZH11 and *smg5*. n = 12 **(D)**, n = 11 **(E)**. **(F-I)**, The panicle length **(F)**, number of primary branches **(G)**, number of secondary branches **(H)**, and grain number per panicle **(I)** of ZH11 and *smg5*. n = 10 **(F-I)**. **(J, K)**, The leaf length **(J)** and leaf width **(K)** of ZH11 and *smg5*. n = 12 **(J, K)**. Values in **(D-K)** are given as mean ± SD. **P < 0.01 compared with ZH11 by Student’s *t*-test. Bars: 10 cm **(A)**, 5 cm **(B)** and 1 cm **(C)**.

Mature *smg5* plants were significantly shorter than ZH11 plants (**Figures 2A, D**). We further measured the length of individual internodes and found that the third to fifth internodes were markedly shorter in *smg5* compared to the wild type (**Supplementary Figures 1A, C**). Additionally, the diameter of the first to fourth internodes was significantly reduced in the *smg5* (**Supplementary Figures 1B, D**). The *smg5* mutant also produced fewer tillers than the wild type (**Figures 2A, E**). In addition, *smg5* panicles were shorter than ZH11 panicles (**Figures 2B, F**). As panicle structure is determined by branch development, we examined panicle branches and found that both primary and secondary branches were significantly reduced in *smg5* (**Figures 2B, G, H**). Consistent with the reduced panicle branching, the grain number per panicle also decreased in the *smg5* mutant (**Figures 2B, I**). The leaves of *smg5* were narrower than those of ZH11 and displayed distinctive dark brown spots, while the leaf length was comparable to that of ZH11 (**Figures 2C, J, K**). These results suggest that *SMG5* also coordinately regulates plant architecture, panicle development, and leaf morphology in rice.

### *SMG5* regulates both cell expansion and cell proliferation in spikelet hulls

**Figure 3:**
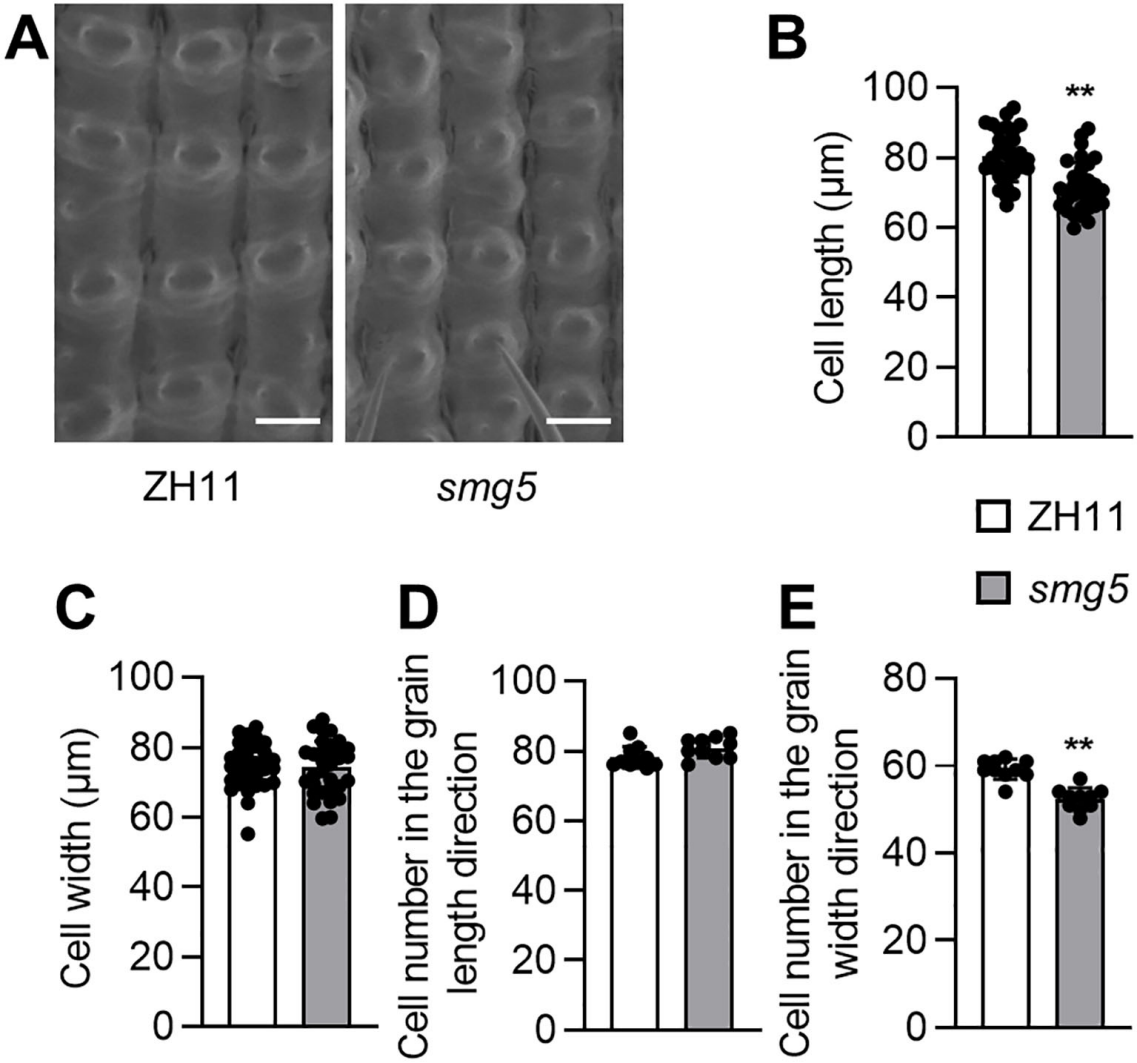
*SMG5* regulates both cell expansion and cell proliferation in spikelet hulls. **(A)** The outer surface of ZH11 and *smg5* spikelet hulls. **(B, C)**, Average length **(B)** and width **(C)** of outer epidermal cells of ZH11 and *smg5* lemmas. n = 32 **(B, C)**. **(D, E)**, The number of outer epidermal cells in the grain-length **(D)** and grain-width **(E)** direction of ZH11 and *smg5*. n = 10 **(D, E)**. Values in **(B-E)** are given as mean ± SD. **P < 0.01 compared with ZH11 by Student’s *t*-test. Bars: 50 μm **(A)**.

The spikelet hull acts as a physical constraint that determines the final grain size in rice. The growth of the spikelet hull is coordinately regulated by the cell proliferation and cell expansion. To investigate the cellular basis of the small-grain phenotype in *smg5*, we examined the epidermal cells of the spikelet hulls. Scanning electron microscopy (SEM) revealed that the outer epidermal cells of *smg5* lemmas were significantly shorter in the longitudinal direction, whereas cell width in the transverse direction remained comparable to that of the wild type (**Figures 3A–C**). Furthermore, cell number quantification revealed no significant difference in cell number along the longitudinal direction between *smg5* and ZH11, but a marked reduction in the transverse direction in the *smg5* mutant (**Figures 3A, D, E**). These results indicate that the reduced grain length in the *smg5* mutant results primarily from impaired cell elongation, whereas the decreased grain width arises from a reduction in cell number. Collectively, these findings demonstrate that *SMG5* regulates grain size by independently regulating cell expansion along the longitudinal direction and cell proliferation along the transverse direction during spikelet hull development.

### Identification of the *SMG5* gene

**Figure 4:**
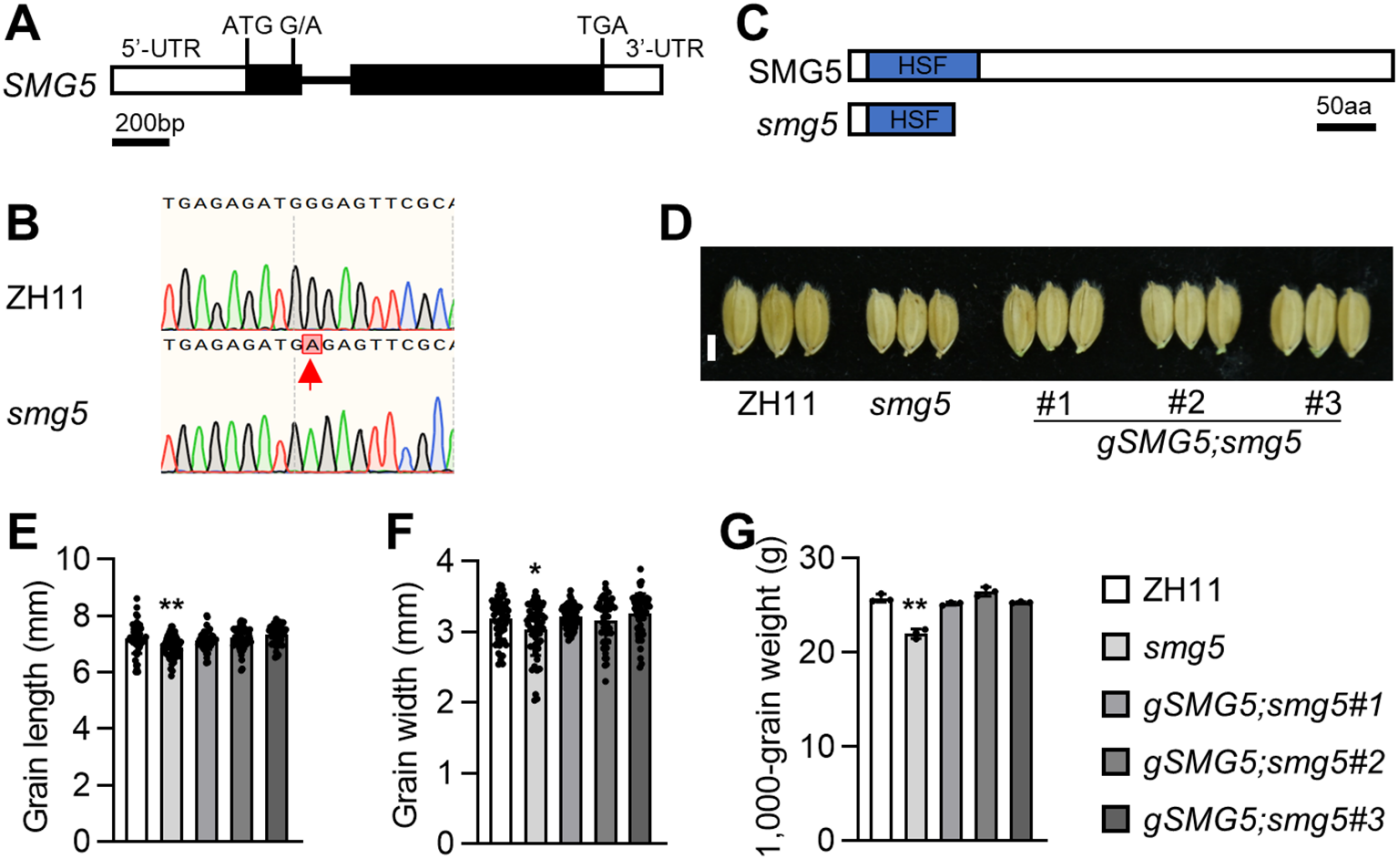
*SMG5* encodes a heat shock transcription factor. **(A)** The *SMG5* gene structure. The black boxes represent the coding sequence, and the white boxes show the 5’ and 3’ untranslated regions. The start codon (ATG) and the stop codon (TAG) are shown. **(B)**, The mutation site of *smg5* was confirmed by sequencing. **(C)**, The schematic diagram of the SMG5 protein and the truncated proteins generated by *smg5* mutation. **(D)**, Mature grains of ZH11, *smg5*, and *gSMG5*;*smg5* transgenic lines. **(E-G)**, Grain length **(E)**, grain width **(F)** and 1,000 grain weight of ZH11, *smg5*, and *gSMG5*;*smg5* transgenic lines. n = 60 **(E-F)**, and n = 3 **(G)**. Values in **(E-G)** are given as mean ± SD. **P < 0.01 compared with ZH11 by Student’s *t*-test. Bars: 3 mm **(D)**.

To identify the causal mutation for the *smg5* phenotype, we employed the MutMap approach (Abe et al., 2012). Genetic analysis of an F_2_ population derived from a cross between *smg5* and the wild-type ZH11 indicated that the small-grain phenotype is controlled by a single recessive locus. We then performed whole-genome sequencing of pooled DNA from F_2_ individuals displaying the small-grain phenotype, alongside sequencing of ZH11 as a reference. Single-nucleotide polymorphism (SNP) analyses were performed as described previously (Fang et al., 2016; Huang et al., 2017). In total, 1186 SNPs and 2095 INDELs were identified between pooled F_2_ individuals with small-grain phenotype and ZH11. The SNP/INDEL-index in the pooled F_2_ plants was calculated in the whole genome. Among them, only the SNP2 variant was located within an exonic region and exhibited an SNP/INDEL-index = 1 (**Supplementary Table 1**). Sanger sequencing confirmed this mutation in the *smg5* mutant (**Figure 4B**). This SNP (G to A) occurs in the first exon of the *LOC_Os05g45410* gene, resulting in a premature stop codon (**Figure 4A**), suggesting that the *LOC_Os05g45410* could be the *SMG5* gene.

To verify that *LOC_Os05g45410* is the *SMG5* gene, we performed a genetic complementation test. The genomic fragment containing the entire *SMG5* coding region, together with a 2000 bp 5’ flanking sequence and a 1000 bp 3’ flanking sequence, was introduced into the *smg5* mutant background. We obtained 10 independent transgenic lines, and the *gSMG5* construct complemented the phenotypes of the *smg5* (**Figures 4D–G**). The grain length, grain width and grain weight of *gSMG5*;*smg5* transgenic plants were comparable with those of ZH11 (**Figures 4D–G**). In addition, the *gSMG5*;*smg5* transgenic plants displayed wild-type plant architecture and leaf morphology (**Supplementary Figure 2**). Collectively, these results demonstrate that *LOC_Os05g45410* is the *SMG5* gene.

The *SMG5* gene encodes a heat shock transcription factor and was previously reported as *SPOTTED LEAF 7* (*SPL7*). Previous studies have established *OsSPL7* as a key regulator of heat stress responses, reactive oxygen species (ROS) homeostasis, and pathogen resistance in rice (Hoang et al., 2019, 2023; Mittal et al., 2009; Yamanouchi et al., 2002), whereas its roles in regulating grain size remained largely unexplored. Accordingly, *smg5* represents a novel allele of *SPL7*. The mutation in *smg5* introduces a premature stop codon, resulting in a truncated protein lacking part of the conserved HSF domain (**Figure 4C**), indicating that the *smg5* is a loss-of-function allele.

### Expression and subcellular localization of SMG5

**Figure 5:**
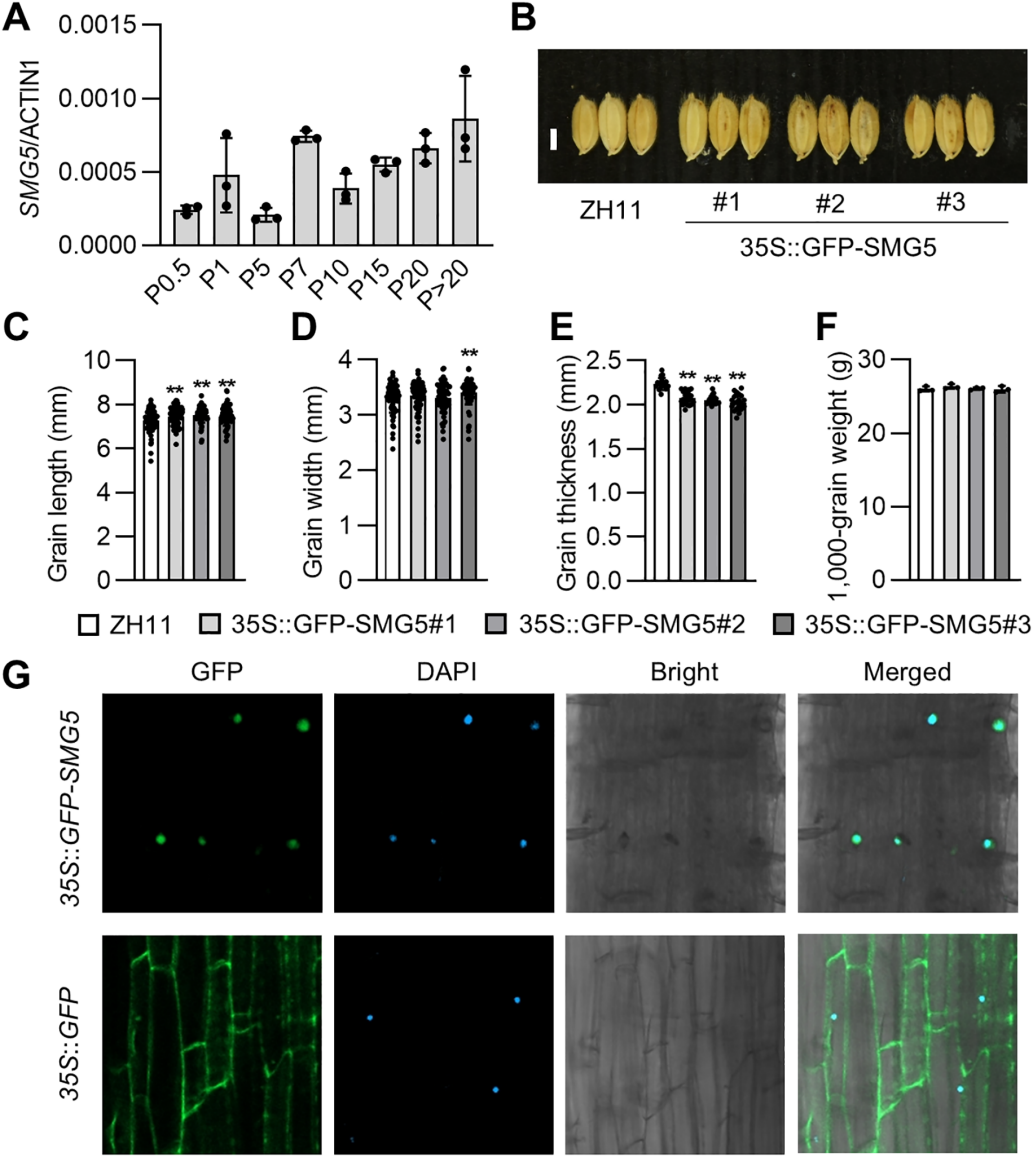
Expression and subcellular localization of SMG5. **(A)** The transcript level of *SMG5* in developing panicles. qRT–PCR was performed with three replicates. **(B)**, Mature grains of the ZH11 and *pro35S:GFP-SMG5* transgenic lines. **(C-F)**, The grain length **(C)**, grain width **(D)**, grain thickness **(E)**, and 1,000 grain weight **(F)** of ZH11 and *pro35S:GFP-SMG5* transgenic lines. n = 85 **(C, D)**, n = 20 **(E)**, and n =3 **(F)**. **(G)**, Subcellular localization of GFP-SMG5 in *pro35S*:GFP-SMG5 coleoptiles cells. The GFP fluorescence of GFP-SMG5 fused protein, DAPI staining, bright field, and their merged images are displayed. Values in **(C-F)** are given as mean ± SD. **P < 0.01 compared with ZH11 by Student’s *t*-test. Bars: 0.3 mm **(B)**.

We analyzed the expression profile of *SMG5* in developing panicles at different stages using quantitative real-time RT-PCR. The expression of *SMG5* was detected throughout panicle development and displayed dynamic changes during different stages (**Figure 5A**). To investigate the subcellular localization of SMG5, we generated *pro35S*:*GFP*-*SMG5* transgenic lines in the ZH11 background. The *pro35S*:*GFP*-*SMG5* transgenic lines exhibited significantly increased grain length, accompanied by reduced grain thickness (**Figures 5B–E**). Consequently, the 1000-grain weight of the transgenic plants was comparable to that of ZH11 (**Figure 5F**), indicating that the GFP-SMG5 fusion protein retains its biological function in regulating grain size (**Figure 5B**). In addition, the *pro35S*:*GFP*-*SMG5* transgenic lines showed no significant differences from ZH11 in other agronomic traits, including plant height, tilling number, leaf length and width, panicle length, primary and secondary branch numbers, and grain number per panicle (**Supplementary Figure 3**). GFP fluorescence was predominantly localized to the nuclei of coleoptiles cells in *pro35S*:*GFP*-*SMG5* plants (**Figure 5G**), indicating that SMG5 is a nuclear-localized protein, consistent with its role as a transcription factor.

### SMG5 directly regulates the expression of *DGS1*

To investigate the molecular mechanism by which *SMG5* regulates grain size, we performed RNA-seq analysis using young panicles from ZH11 and the *smg5* mutant. A total of 2,133 differentially expressed genes (DEGs) were identified, including 1,110 upregulated and 1,033 downregulated genes in *smg5* (**Supplementary Figure 4A**, **Supplementary Data 1**). Notably, the expression of several previously characterized grain-size regulators was altered in the *smg5* mutant, including *D11*, *OsCEP6*, *UBC45*, *OsMADS56*, *OsATG13a*, *OML4*, and *OsDGS1* (Li et al., 2023; Liu et al., 2023; Lyu et al., 2020; Sui et al., 2016; Tanabe et al., 2005; Zhan et al., 2022; Zhu et al., 2021)(**Supplementary Figure 4B**, **Supplementary Data 1**). The qRT-PCR results confirmed the expression trends observed in the RNA-seq data, supporting the reliability of our transcriptomic profiling (**Supplementary Figures 4C–H**). GO enrichment analysis revealed that these DEGs were significantly enriched in biological processes related to photosynthesis, chromatin remodeling, and plant organ development (**Supplementary Figure 5**).

**Figure 6:**
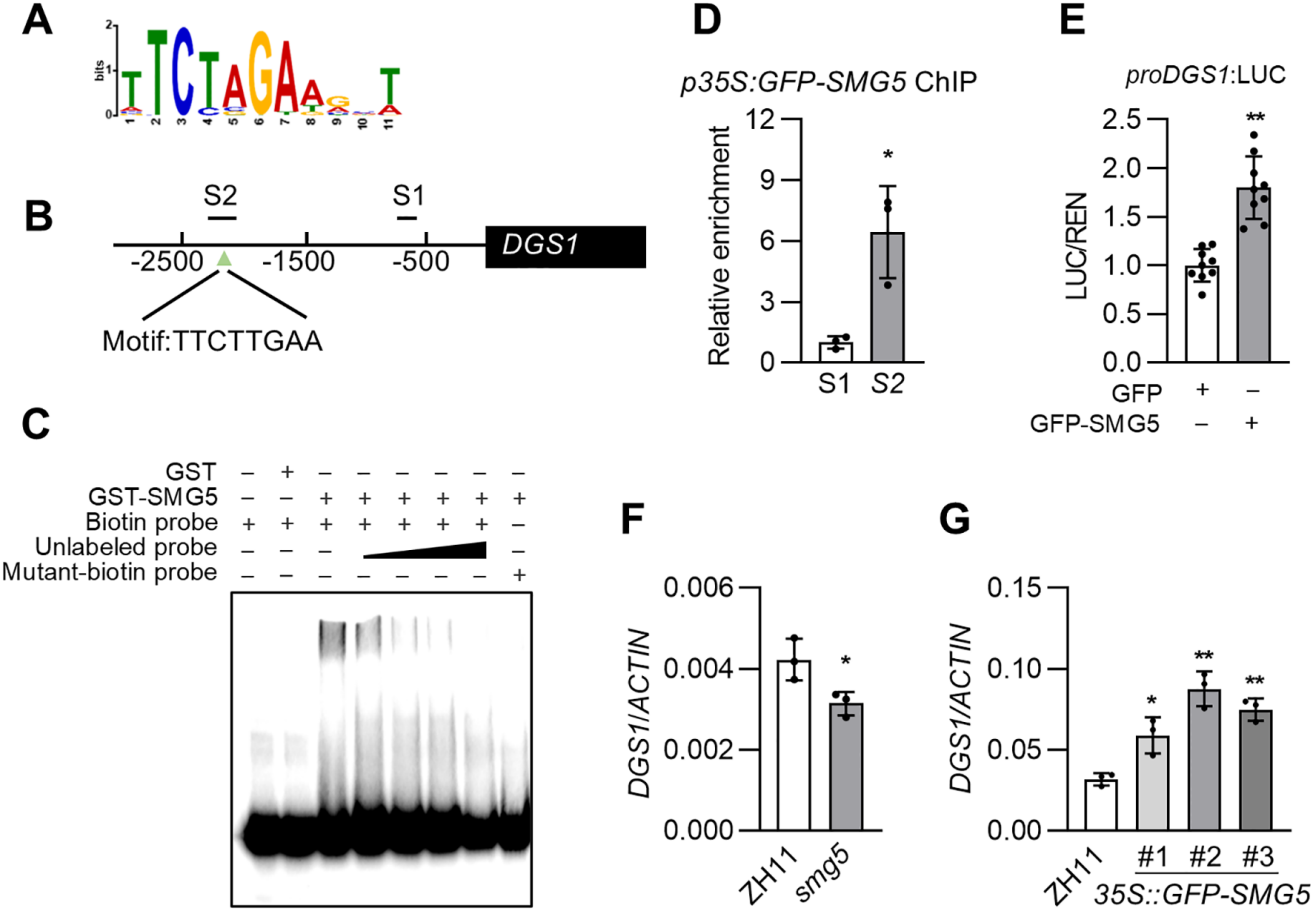
SMG5 binds to specific cis-element and regulates target gene expression. **(A)** The enriched motif in SMG5-binding sites *in vitro*. The SMG5-binding sites were identified using the DAP-seq method. **(B)** The S2 fragment in the 2.5 kb promoter region of *SMG5* contains an TTCTTGAA sequence, but S1 does not. **(C)** EMSA showed that the GST–SMG5 fusion protein specifically binds to probes containing TTCTTGAA sequences but not the mutant probes. **(D)** ChIP–qPCR validation of SMG5 binding at the promoter regions of *DGS1*. The data are presented as fold enrichment of detected sites relative to the negative control (S1) immunoprecipitation signal. For the negative control, we selected regions without binding motif (S1) in the promoter regions of *DGS1*. Three replicates were performed. **(E)** The dual-luciferase assays shown that SMG5 activates the transcriptional activation from *DGS1* promoter in rice protoplasts. n = 9. **(F)** The relative expression levels of *DGS1* in young panicles of ZH11 and *smg5.* **(G)** The relative expression levels of DGS1 in ZH11 and 35S::GFP−SMG5 seedlings. Values in **(D-G)** are given as mean ± SD. *P < 0.05 or **P < 0.01 compared with ZH11 (**F** and **G**),the negative control S1 **(D)**,or the GFP **(E)**.

To identify the DNA-binding motif of SMG5, we performed DNA affinity purification sequencing (DAP-seq) using a GST-SMG5 fusion protein and a genomic DNA library derived from rice panicles. Analysis of the DAP-seq data revealed that approximately 49% of the SMG5 binding peaks were located within promoter regions (**Supplementary Figures 6A, B**). We then identified the SMG5-binding motifs using the MEME-chip method, among which TTCTAGAA was the most significantly enriched (**Figures 6A**; **Supplementary Figure 6C**). To validate the putative binding sequences of SMG5 identified by DAP-seq, we performed electrophoretic mobility shift assay (EMSA) *in vitro*. As shown in **Supplementary Figures 6D**, the GST-SMG5 fusion protein specifically bound to probes containing the TTCTAGAA motif, but failed to bind to probes carrying a mutated version of the motif. The specificity of this binding was further confirmed by competition assays, in which increasing amounts of unlabeled wild-type probe competitively reduced the binding signal in a dose-dependent manner (**Supplementary Figure 6D**).

To identify direct downstream targets of SMG5, we screened the promoter regions of seed-size regulator genes from our RNA-seq data for the presence of SMG5-binding motif TTCTAGAA, allowing for a single-nucleotide mismatch to accommodate potential sequence variation. This analysis revealed candidate binding sites in the promoters of several genes, including *DGS1*. Previous studies have reported that loss-of-function mutations in DGS1 resulted in smaller grains in rice (Li et al., 2023). To functionally validate this predicted interaction, we synthesized a short DNA probe encompassing the candidate site within the *DGS1* promoter containing the sequence TTCTTGAA (a single-nucleotide difference from the binding motif identified by DAP-seq) (**Figure 6B**). Electrophoretic mobility shift assay (EMSA) confirmed that that SMG5 specifically bound to this probe, whereas a mutated probe in which the core TTCTTGAA sequence was disrupted failed to exhibit detectable binding (**Figure 6C**). Subsequent ChIP-qPCR analysis confirmed specific enrichment of SMG5 at the S2 region of the *DGS1* promoter, which contains the TTCTTGAA sequences (**Figure 6D**). To test whether OsSMG5 directly regulates the expression of *DGS1*, we performed dual-luciferase transactivation assays in ZH11 protoplasts. Compared with the empty *35S*:*GFP* control, overexpression of *OsSMG5* under the control of the *35S* promoter significantly induced LUC expression driven by the *DGS1* promoter (**Figure 6E**). These results consistent with the role of SMG5 as a transcriptional activator (Hoang et al., 2023). The expression of *DGS1* was significantly reduced in the *smg5* mutant (**Figure 6F**), while it was significantly increased in the *pro35S:GFP-SMG5* overexpression lines (**Figure 6G**). Collectively, these results demonstrate that SMG5 directly binds to the *DGS1* promoter and activates its transcription to regulate grain size.

## Discussion

Grain size and weight are crucial determinants of rice yield, yet the genetic and molecular mechanisms controlling these traits remain incompletely understood. In this study, we identify SMG5/SPL7, a heat shock transcription factor, as a key regulator of grain size that coordinates both cell expansion and cell proliferation in spikelet hulls. Our findings demonstrate that *SMG5* directly regulates the expression of *DGS1*, thereby establishing a transcriptional module that links stress-associated transcription factors to hormone-mediated grain development.

*SMG5* was identified as an allele of *OsHsfA4d*/*SPL7*, a gene previously characterized for its roles in heat stress responses, reactive oxygen species (ROS) homeostasis, and pathogen resistance in rice (Fang et al., 2025; Hoang et al., 2019, 2023; Mittal et al., 2009; Yamanouchi et al., 2002). While overexpression of *OsHsfA4d*/*SPL7* enhances heat tolerance (Fang et al., 2025), both knockdown and overexpression of this gene have been shown to potentiate defense response against bacterial pathogens (Hoang et al., 2019). Our study extends these findings by revealing an additional role for SMG5 in regulating grain size through the SMG5-DGS1 module. *DGS1*, also known as *TT3.1*, has been recently reported to interact with SMALL GRAIN 3 (SMG3) to form an ERAD-related E2–E3 enzyme pair that modulates grain size and weight via the brassinosteroid (BR) signaling pathway (Li et al., 2023; Zhang et al., 2022). Previous studies have shown that OsBZR1, a key transcription factor in brassinosteroid signaling, directly binds to the *DGS1* promoter and activates its expression (Zhu et al., 2021). Our finding that SMG5 also directly activates *DGS1* suggests that this gene may serve as an integration node for multiple regulatory inputs. Whether SMG5 and OsBZR1 function independently, cooperatively, or competitively in regulating *DGS1* expression remains to be determined. It is possible that these two transcription factors respond to different upstream signals, with SMG5 responding to stress-associated cues and OsBZR1 responding to BR signaling, and converge on *DGS1* to coordinate growth and stress responses. Notably, *DGS1*/*TT3.1* also contributes to thermotolerance; under field heat stress conditions, overexpression of *TT3.1* significantly increases rice yield, whereas it has no negative impact on yield traits under normal conditions. Therefore, by directly regulating *DGS1/TT3.1* expression, SMG5 may participate in coordinating both heat stress adaptation and grain development. These findings suggest a dual functionality for SMG5 in mediating stress responses and developmental processes, reflecting an evolutionary adaptation in which plants utilize stress-responsive regulators to fine-tune growth under fluctuating environmental conditions.

The *smg5* mutant exhibited small and light grains with reduced length, width, and thickness (**Figure 1**), whereas *SMG5* overexpression resulted in increased grain size (**Figure 5B**). Cellular analysis revealed that *SMG5* regulates grain size by independently controlling cell expansion in the grain-length direction and cell proliferation in the grain-width direction during spikelet hull development (**Figure 3**). In addition to its effects on grain traits, the *smg5* mutant displayed pleiotropic defects, including reductions in plant height, tiller number, panicle branching, and grain number per panicle (**Figure 2**), suggesting that *SMG5* plays a broader role in coordinating plant growth and development in rice. Interestingly, we observed reduced grain thickness in both the *smg5* mutant and the *35S:GFP-SMG5* overexpression lines. This unexpected observation may reflect a dosage-dependent effect of *SMG5*. It is possible that constitutive high-level expression driven by the *35S* promoter disrupts the precise spatiotemporal regulation required for balanced grain development. Similar phenomena have been reported in other studies. For instance, both overexpression and downregulation of *OsWOX11* results in pleiotropic developmental defects, including dwarfism and reduced yield (Zhang et al., 2025; Zhao et al., 2009). Likewise, both loss-of-function and overexpression of *SDG711* lead to a decreased number of sclerenchymatous cells in the flag leaf (Lu et al., 2024). These findings support the notion that optimal expression levels of developmental regulators such as *SMG5* are critical for normal grain morphogenesis.

DAP-seq combined with EMSA analyses revealed that SMG5 directly binds to both the TTCTAGAA and TTCTTGAA motif (**Figure 6C**; **Supplementary Figures 6C, D**). SMG5 belongs to the type A heat shock transcription factors, a subclass that is typically associated with transcriptional activation (Hoang et al., 2023). Consistent with this classification, our results demonstrate that SMG5 associates to the promoter region of *DGS1* and activates its transcription (**Figures 6B–F**). Our RNA-seq analysis revealed that multiple genes involved in known grain size regulatory pathways are differentially expressed in the *smg5* mutant. Notably, genes related to BR signaling, including *D11* and *OML4*, exhibited significantly altered expression levels (**Supplementary Figure 4**). *D11* encodes a cytochrome P450 enzyme involved in BR biosynthesis, while *OML4* has been implicated in BR-mediated growth regulation (Lyu et al., 2020; Tanabe et al., 2005). The altered expression of these genes in *smg5* suggests that SMG5 may influence grain size, at least in part, by modulating the BR signaling pathway. Furthermore, we observed differential expression of genes with established roles in distinct cellular processes, which may explain how *SMG5* coordinates the opposing processes of cell expansion and proliferation. Specifically, genes such as *D11*, *DGS1*, *OML4*, and *MADS56* have been reported to influence cell expansion, whereas *OsATG13a*, an autophagy-related gene, has been implicated in regulating cell proliferation during grain development (Li et al., 2023; Liu et al., 2023; Lyu et al., 2020; Tanabe et al., 2005; Zhan et al., 2022). The expression of all these genes was altered in the *smg5* mutant, suggesting that *SMG5* may coordinate the balance between cell expansion and proliferation through the differential regulation of distinct downstream genes.

Notably, although *OsSMG5* overexpression increases grain length, it reduces grain thickness, resulting in no change in the final 1000-grain weight. In addition, the tiller number and grain number per panicle show little difference compared with ZH11, suggesting that overall yield is likely unchanged in normal conditions (**Figure 5**; **Supplementary Figure 3**). These results suggest that SMG5 may have potential utility for rice yield improvement under high-temperature conditions, given its dual role in regulating *DGS1*/*TT3.1*, a gene known to enhance thermotolerance. In summary, this study establishes SMG5 as a transcriptional regulator that coordinates grain size through both cell expansion and cell proliferation pathways. The identification of its direct target *DGS1* and its integration with BR signaling provides mechanistic insights into grain size regulation. Future studies exploring natural variation in *SMG5*, identifying additional downstream targets, and elucidating its regulatory networks may reveal valuable genetic resources for crop improvement under increasingly challenging environmental conditions.

## Data Availability

The original contributions presented in the study are publicly available. This data can be found in the Genome Sequence Archive database (https://ngdc.cncb.ac.cn/gsa/, accession number, CRA040066 (DAP-seq data) and CRA040055 (RNA-seq data) in National Genomics Data Center, China National Center for Bioinformation.

## References

[B1] Abe A. Kosugi S. Yoshida K. Natsume S. Takagi H. Kanzaki H. . (2012). Genome sequencing reveals agronomically important loci in rice using MutMap. Nat. Biotechnol. 30, 174–178. doi: 10.1038/nbt.2095. PMID: 22267009

[B2] Achary R. K. Kamble N. U. Gautam S. Hazra A. Varshney V. Mahawar S. . (2025). The rice heat shock transcription factor OsHSFC1b increases seed weight, size, and vigor, but its function is disrupted by isoaspartyl modification. Plant J. 123, e70365. doi: 10.1111/tpj.70365. PMID: 40719514

[B3] Bartlett A. O’Malley R. C. Huang S. C. Galli M. Nery J. R. Gallavotti A. . (2017). Mapping genome-wide transcription-factor binding sites using DAP-seq. Nat. Protoc. 12, 1659–1672. doi: 10.1038/nprot.2017.055. PMID: 28726847 PMC5576341

[B4] Duan P. Ni S. Wang J. Zhang B. Xu R. Wang Y. . (2015). Regulation of OsGRF4 by OsmiR396 controls grain size and yield in rice. Nat. Plants 2, 15203. doi: 10.1038/nplants.2015.203. PMID: 27250749

[B5] Duan P. Rao Y. Zeng D. Yang Y. Xu R. Zhang B. . (2014). SMALL GRAIN 1, which encodes a mitogen-activated protein kinase kinase 4, influences grain size in rice. Plant J. 77, 547–557. doi: 10.1111/tpj.12405, PMID: 24320692

[B6] Duan P. Xu J. Zeng D. Zhang B. Geng M. Zhang G. . (2017). Natural variation in the promoter of GSE5 contributes to grain size diversity in rice. Mol. Plant 10, 685–694. doi: 10.1016/j.molp.2017.03.009. PMID: 28366824

[B7] Fang Y. Liao H. Wei Y. Yin J. Cha J. Liu X. . (2025). OsCDPK24 and OsCDPK28 phosphorylate heat shock factor OsHSFA4d to orchestrate abiotic and biotic stress responses in rice. Nat. Commun. 16, 6485. doi: 10.1038/s41467-025-61827-6. PMID: 40659645 PMC12260056

[B8] Fang N. Xu R. Huang L. Zhang B. Duan P. Li N. . (2016). SMALL GRAIN 11 controls grain size, grain number and grain yield in rice. Rice (N Y) 9, 64. doi: 10.1186/s12284-016-0136-z. PMID: 27900723 PMC5127926

[B9] Guo T. Chen K. Dong N. Q. Shi C. L. Ye W. W. Gao J. P. . (2018). GRAIN SIZE AND NUMBER1 negatively regulates the OsMKKK10-OsMKK4-OsMPK6 cascade to coordinate the trade-off between grain number per panicle and grain size in rice. Plant Cell 30, 871–888. doi: 10.1105/tpc.17.00959. PMID: 29588389 PMC5973843

[B10] Hao Y.-J. Song Q.-X. Chen H.-W. Zou H.-F. Wei W. Kang X.-S. . (2010). Plant NAC-type transcription factor proteins contain a NARD domain for repression of transcriptional activation. Planta 232, 1033–1043. doi: 10.1007/s00425-010-1238-2. PMID: 20683728

[B11] Hoang T. V. Vo K. T. X. Rahman M. M. Choi S. H. Jeon J. S. (2019). Heat stress transcription factor OsSPL7 plays a critical role in reactive oxygen species balance and stress responses in rice. Plant Sci. 289, 110273. doi: 10.1016/j.plantsci.2019.110273. PMID: 31623772

[B12] Hoang T. V. Vo K. T. X. Rahman M. M. Zhong R. Lee C. Ketudat Cairns J. R. . (2023). SPOTTED-LEAF7 targets the gene encoding beta-galactosidase9, which functions in rice growth and stress responses. Plant Physiol. 193, 1109–1125. doi: 10.1093/plphys/kiad359. PMID: 37341542 PMC10517187

[B13] Hu J. Wang Y. Fang Y. Zeng L. Xu J. Yu H. . (2015). A rare allele of GS2 enhances grain size and grain yield in rice. Mol. Plant 8, 1455–1465. doi: 10.1016/j.molp.2015.07.002. PMID: 26187814

[B14] Huang K. Wang D. Duan P. Zhang B. Xu R. Li N. . (2017). WIDE AND THICK GRAIN 1, which encodes an otubain-like protease with deubiquitination activity, influences grain size and shape in rice. Plant J. 91, 849–860. doi: 10.1111/tpj.13613. PMID: 28621888

[B15] Huang K. Wang Y. Li Y. Zhang B. Zhang L. Duan P. . (2024). Modulation of histone acetylation enables fully mechanized hybrid rice breeding. Nat. Plants 10, 954–970. doi: 10.1038/s41477-024-01720-0. PMID: 38831046

[B16] Li Y. Fan C. Xing Y. Jiang Y. Luo L. Sun L. . (2011). Natural variation in GS5 plays an important role in regulating grain size and yield in rice. Nat. Genet. 43, 1266–1269. doi: 10.1038/ng.977. PMID: 22019783

[B17] Li S. Gao F. Xie K. Zeng X. Cao Y. Zeng J. . (2016). The OsmiR396c-OsGRF4-OsGIF1 regulatory module determines grain size and yield in rice. Plant Biotechnol. J. 14, 2134–2146. doi: 10.1111/pbi.12569. PMID: 27107174 PMC5095787

[B18] Li N. Li Y. (2016). Signaling pathways of seed size control in plants. Curr. Opin. Plant Biol. 33, 23–32. doi: 10.1016/j.pbi.2016.05.008. PMID: 27294659

[B19] Li N. Xu R. Li Y. (2019). Molecular networks of seed size control in plants. Annu. Rev. Plant Biol. 70, 435–463. doi: 10.1146/annurev-arplant-050718-095851. PMID: 30795704

[B20] Li J. Zhang B. Duan P. Yan L. Yu H. Zhang L. . (2023). An endoplasmic reticulum-associated degradation-related E2-E3 enzyme pair controls grain size and weight through the brassinosteroid signaling pathway in rice. Plant Cell 35, 1076–1091. doi: 10.1093/plcell/koac364. PMID: 36519262 PMC10015164

[B21] Liu J. Chen J. Zheng X. Wu F. Lin Q. Heng Y. . (2017). GW5 acts in the brassinosteroid signalling pathway to regulate grain width and weight in rice. Nat. Plants 3, 17043. doi: 10.1038/nplants.2017.43. PMID: 28394310

[B22] Liu S. Hua L. Dong S. Chen H. Zhu X. Jiang J. . (2015). OsMAPK6, a mitogen-activated protein kinase, influences rice grain size and biomass production. Plant J. 84, 672–681. doi: 10.1111/tpj.13025. PMID: 26366992

[B23] Liu Z. Yang Q. Wu P. Li Y. Lin Y. Liu W. . (2023). Dynamic monitoring of TGW6 by selective autophagy during grain development in rice. New Phytol. 240, 2419–2435. doi: 10.1111/nph.19271. PMID: 37743547

[B24] Liu A. L. Zou J. Liu C. F. Zhou X. Y. Zhang X. W. Luo G. Y. . (2013). Over-expression of OsHsfA7 enhanced salt and drought tolerance in transgenic rice. BMB Rep. 46, 31–36. doi: 10.5483/bmbrep.2013.46.1.090. PMID: 23351381 PMC4133825

[B25] Lu J. Jiang Z. Chen J. Xie M. Huang W. Li J. . (2024). SET DOMAIN GROUP 711-mediated H3K27me3 methylation of cytokinin metabolism genes regulates organ size in rice. Plant Physiol. 194, 2069–2085. doi: 10.1093/plphys/kiad568. PMID: 37874747

[B26] Lyu J. Wang D. Duan P. Liu Y. Huang K. Zeng D. . (2020). Control of grain size and weight by the GSK2-LARGE1/OML4 pathway in rice. Plant Cell 32, 1905–1918. doi: 10.1105/tpc.19.00468. PMID: 32303659 PMC7268794

[B27] Mao H. Sun S. Yao J. Wang C. Yu S. Xu C. . (2010). Linking differential domain functions of the GS3 protein to natural variation of grain size in rice. Proc. Natl. Acad. Sci. U.S.A. 107, 19579–19584. doi: 10.1073/pnas.1014419107. PMID: 20974950 PMC2984220

[B28] Mittal D. Chakrabarti S. Sarkar A. Singh A. Grover A. (2009). Heat shock factor gene family in rice: genomic organization and transcript expression profiling in response to high temperature, low temperature and oxidative stresses. Plant Physiol. Biochem. 47, 785–795. doi: 10.1016/j.plaphy.2009.05.003. PMID: 19539489

[B29] Schmidt R. Schippers J. H. Welker A. Mieulet D. Guiderdoni E. Mueller-Roeber B. (2012). Transcription factor OsHsfC1b regulates salt tolerance and development in Oryza sativa ssp. japonica. AoB Plants 2012, pls011. doi: 10.1093/aobpla/pls011. PMID: 22616023 PMC3357053

[B30] Shim D. Hwang J. U. Lee J. Lee S. Choi Y. An G. . (2009). Orthologs of the class A4 heat shock transcription factor HsfA4a confer cadmium tolerance in wheat and rice. Plant Cell 21, 4031–4043. doi: 10.1105/tpc.109.066902. PMID: 20028842 PMC2814514

[B31] Si L. Chen J. Huang X. Gong H. Luo J. Hou Q. . (2016). OsSPL13 controls grain size in cultivated rice. Nat Genet. 48, 447–456. 26950093 10.1038/ng.3518

[B32] Song X. J. Huang W. Shi M. Zhu M. Z. Lin H. X. (2007). A QTL for rice grain width and weight encodes a previously unknown RING-type E3 ubiquitin ligase. Nat. Genet. 39, 623–630. doi: 10.1038/ng2014. PMID: 17417637

[B33] Sui Z. Wang T. Li H. Zhang M. Li Y. Xu R. . (2016). Overexpression of peptide-encoding OsCEP6.1 results in pleiotropic effects on growth in rice (O. sativa). Front. Plant Sci. 7. doi: 10.3389/fpls.2016.00228. PMID: 26973672 PMC4773640

[B34] Sun P. Zhang W. Wang Y. He Q. Shu F. Liu H. . (2016). OsGRF4 controls grain shape, panicle length and seed shattering in rice. J. Integr. Plant Biol. 58, 836–847. doi: 10.1111/jipb.12473. PMID: 26936408 PMC5089622

[B35] Tanabe S. Ashikari M. Fujioka S. Takatsuto S. Yoshida S. Yano M. . (2005). A novel cytochrome P450 is implicated in brassinosteroid biosynthesis via the characterization of a rice dwarf mutant, dwarf11, with reduced seed length. Plant Cell 17, 776–790. doi: 10.1105/tpc.104.024950. PMID: 15705958 PMC1069698

[B36] Wang S. Wu K. Qian Q. Liu Q. Li Q. Pan Y. . (2017). Non-canonical regulation of SPL transcription factors by a human OTUB1-like deubiquitinase defines a new plant type rice associated with higher grain yield. Cell Res. 27, 1142–1156. doi: 10.1038/cr.2017.98. PMID: 28776570 PMC5587855

[B37] Wang S. Wu K. Yuan Q. Liu X. Liu Z. Lin X. . (2012). Control of grain size, shape and quality by OsSPL16 in rice. Nat. Genet. 44, 950–954. doi: 10.1038/ng.2327. PMID: 22729225

[B38] Xiang J. Ran J. Zou J. Zhou X. Liu A. Zhang X. . (2013). Heat shock factor OsHsfB2b negatively regulates drought and salt tolerance in rice. Plant Cell Rep. 32, 1795–1806. doi: 10.1007/s00299-013-1492-4. PMID: 23949687

[B39] Xing Y. Zhang Q. (2010). Genetic and molecular bases of rice yield. Annu. Rev. Plant Biol. 61, 421–442. doi: 10.1146/annurev-arplant-042809-112209. PMID: 20192739

[B40] Xu R. Duan P. Yu H. Zhou Z. Zhang B. Wang R. . (2018a). Control of grain size and weight by the OsMKKK10-OsMKK4-OsMAPK6 signaling pathway in rice. Mol. Plant 11, 860–873. doi: 10.1016/j.molp.2018.04.004. PMID: 29702261

[B41] Xu R. Yu H. Wang J. Duan P. Zhang B. Li J. . (2018b). A mitogen-activated protein kinase phosphatase influences grain size and weight in rice. Plant J. 95, 937–946. doi: 10.1111/tpj.13971. PMID: 29775492

[B42] Yamanouchi U. Yano M. Lin H. Ashikari M. Yamada K. (2002). A rice spotted leaf gene, Spl7, encodes a heat stress transcription factor protein. Proc. Natl. Acad. Sci. U.S.A. 99, 7530–7535. doi: 10.1073/pnas.112209199. PMID: 12032317 PMC124274

[B43] Zhan P. Ma S. Xiao Z. Li F. Wei X. Lin S. . (2022). Natural variations in grain length 10 (GL10) regulate rice grain size. J. Genet. Genomics 49, 405–413. doi: 10.1016/j.jgg.2022.01.008. PMID: 35151907

[B44] Zhang Y. Su J. Duan S. Ao Y. Dai J. Liu J. . (2011). A highly efficient rice green tissue protoplast system for transient gene expression and studying light/chloroplast-related processes. Plant Methods 7, 30. doi: 10.1186/1746-4811-7-30. PMID: 21961694 PMC3203094

[B45] Zhang T. Xiang Y. Ye M. Yuan M. Xu G. Zhou D. X. . (2025). The uORF-HsfA1a-WOX11 module controls crown root development in rice. New Phytol. 247. doi: 10.3389/fpls.2018.00523. PMID: 40396436

[B46] Zhang H. Zhou J.-F. Kan Y. Shan J.-X. Ye W.-W. Dong N.-Q. . (2022). A genetic module at one locus in rice protects chloroplasts to enhance thermotolerance. Science 376, 1293–1300. doi: 10.1126/science.abo5721. PMID: 35709289

[B47] Zhao Y. Hu Y. Dai M. Huang L. Zhou D. X. (2009). The WUSCHEL-related homeobox gene WOX11 is required to activate shoot-borne crown root development in rice. Plant Cell 21, 736–748. doi: 10.1105/tpc.108.061655. PMID: 19258439 PMC2671696

[B48] Zhu X. Zhang S. Chen Y. Mou C. Huang Y. Liu X. . (2021). Decreased grain size1, a C3HC4-type RING protein, influences grain size in rice (Oryza sativa L.). Plant Mol. Biol. 105, 405–417. doi: 10.1007/s11103-020-01096-7. PMID: 33387175

[B49] Zuo J. Li J. (2014). Molecular genetic dissection of quantitative trait loci regulating rice grain size. Annu. Rev. Genet. 48, 99–118. doi: 10.1146/annurev-genet-120213-092138. PMID: 25149369

