# Supplementary material for: SMALL GRAIN 5 encodes a heat shock transcription factor controlling grain size and plant architecture in rice
Source: Front Plant Sci. 2026 Apr 1;17:1783348. doi: 10.3389/fpls.2026.1783348 (PMC13079658; doi:10.3389/fpls.2026.1783348)
Supplement: Supplementary file 1 [file Table1.pdf]

Supplementary table 1. Identification of the smg5 mutation using the MutMap approach.

No.	Chromosome	Position	Reference	Alteration	SNP/INDEL-index	Gene	Note
SNP1	Chr5	24414752	C	A	0.33	—	intergenic
INDEL1	Chr5	25510969	A	AT	0.42	LOC_Os05g43760	upstream
SNP2	Chr5	26415304	G	A	1	LOC_Os05g45410	Stop gained
SNP3	Chr5	27907313	T	C	0.59	LOC_Os05g48520	upstream

Genomic DNAs from F_2 plants with small grains were pooled for the whole genome re-sequencing, and ZH11 was also sequenced as a control. The SNP2 variant was found in the exonic region of the *LOC_Os05g45410* gene, which leads to a premature stop codon. The other SNP/INDELs were located in the intergenic or upstream regions of the genes.

Supplementary Table 2 Primers used in this study

Name	Primers sequence
primers used for constructs	
C99-gSMG5-F	TGACCATGATTACGAATTCGAGCTCATTGGACATATAGCCGATTGTTTAAACC
C99-gSMG5-R	TAAAACGACGGCCAGTGCCAAGCTTACCTTTGAAATGTAGATCTGTGCAC
C43-SMG5-F	TGAACTATACAAAGGCGCGCCAATGGAGAGTTCCAACCTGGG
C43-SMG5-R	CGCTCTAGAACTAGTTAATTAATCAGGTATGCAGAGTCTGCT
DGS1PRO-F4	GCAGCCCGGGGGATCCAAGTTTCTCTCCTCGGCAGC
DGS1PRO-R4	TTGGCGTCTTCCATGGGGGAGACAAAAGTGAGGCGA
GST-SMG5-F	CCGCGTGGATCCCCGGAATTCATGGAGAGTTCCAACCTGGG
GST-SMG5-R	GATGCGGCCGCTCGAGTCGACTCAGGTATGCAGAGTCTGCT
primers used for quantitative real-time PCR	
SMG5-qPCR-F	AGCTCAACACCTACGGTTTC
SMG5-qPCR-R	CGTTTATCTGGTTCTGGAGG
ACTIN1-qPCR-F	TGCTATGTACGTCGCCATCCAG
ACTIN1-qPCR-R	AATGAGTAACCACGCTCCGTCA
LARGE1-qPCR-F	CGTGCGGGGATTTTGGTTTT
LARGE1-qPCR-R	TCCAACTTGCCTCTCTGTGG
UBC45-qPCR-F	CAACCATCATGGAGTGTGCG
UBC45-qPCR-R	AGTCCAACGAACCCAATGCC
d11-qPCR-F	TCACTGCTCCAGGTATGGGA
d11-qPCR-R	GCACTGAAACAGCCTCTCCT
OsCEP6.1-qPCR-F	AGGAAGCATGCCAATGACGA
OsCEP6.1-qPCR-R	ACGCATACGATCGAGAGCTG
OsMADS56-qPCR-F	TGGAGCAGCAGATAGCCAAG
OsMADS56-qPCR-R	CAAGGTTGCGATGCTTTCCG
OsATG13a-qPCR-F	CCACGGCTTGGGTTTTCAAG
OsATG13a-qPCR-R	CTTTCCACCACTGCTCCCTG
DGS1-qPCR-F	ACAGCCTACTCCTGGTGGATT
DGS1-qPCR-R	ATTGAATGGCAAGGAGCCCA
primers used for EMSA	
WT-F-5'biotin	GCTAACGTTAGCATGCTAATTCTAGAATACGCTAACGTAGCATTAAG
WT-F	GCTAACGTTAGCATGCTAATTCTAGAATACGCTAACGTAGCATTAAG
WT-R	CTTAATGCTACGTTAGCGTATCTAGAATTAGCATGCTAACGTTAGC
M-F-5'biotin	GCTAACGTTAGCATGCTAATGTCGATTACGCTAACGTAGCATTAAG
M-F	GCTAACGTTAGCATGCTAATGTCGATTACGCTAACGTAGCATTAAG
M-R	CTTAATGCTACGTTAGCGTAATCGACATTAGCATGCTAACGTTAGC
DGS1-F-5bio	GTTGTTGTTCTTCTTCTGCTTCTTGAAGTTGGTGGTCTGCTGAGCTTTG
DGS1-F-np	GTTGTTGTTCTTCTTCTGCTTCTTGAAGTTGGTGGTCTGCTGAGCTTTG
DGS1-R	CAAAGCTCAGCAGACCACCAACTTCAAGAAGCAGAAGAAGAACAACAAC
mDGS1-F-5bio	GTTGTTGTTCTTCTTCTGCAAAAAAAAGTTGGTGGTCTGCTGAGCTTTG
mDGS1-F-np	GTTGTTGTTCTTCTTCTGCAAAAAAAAGTTGGTGGTCTGCTGAGCTTTG
mDGS1-R	CAAAGCTCAGCAGACCACCAACTTTTTTTTGCAGAAGAAGAACAACAAC
primers used for ChIP-qPCR	
Actin1-CQ-F	TGCGTGTCCTCTTCCTCATCT
Actin1-CQ-R	AAGGCGAATGAAGCGAAAAG
DGS1-CQ-F	TGGCCACACACAAAGCAAGT
DGS1-CQ-R	CTCCGTTGAGGGGTTGATTG
DGS1-NQ-F	GGTTAATGACCCCGGAAGCA
DGS1-NQ-R	GAGGGAGGAGGACGACCAG
