# Supplementary material for: SMALL GRAIN 5 encodes a heat shock transcription factor controlling grain size and plant architecture in rice
Source: Front Plant Sci. 2026 Apr 1;17:1783348. doi: 10.3389/fpls.2026.1783348 (PMC13079658; doi:10.3389/fpls.2026.1783348)
Supplement: Supplementary file 2 [file Presentation1.pptx]

## Slide 1
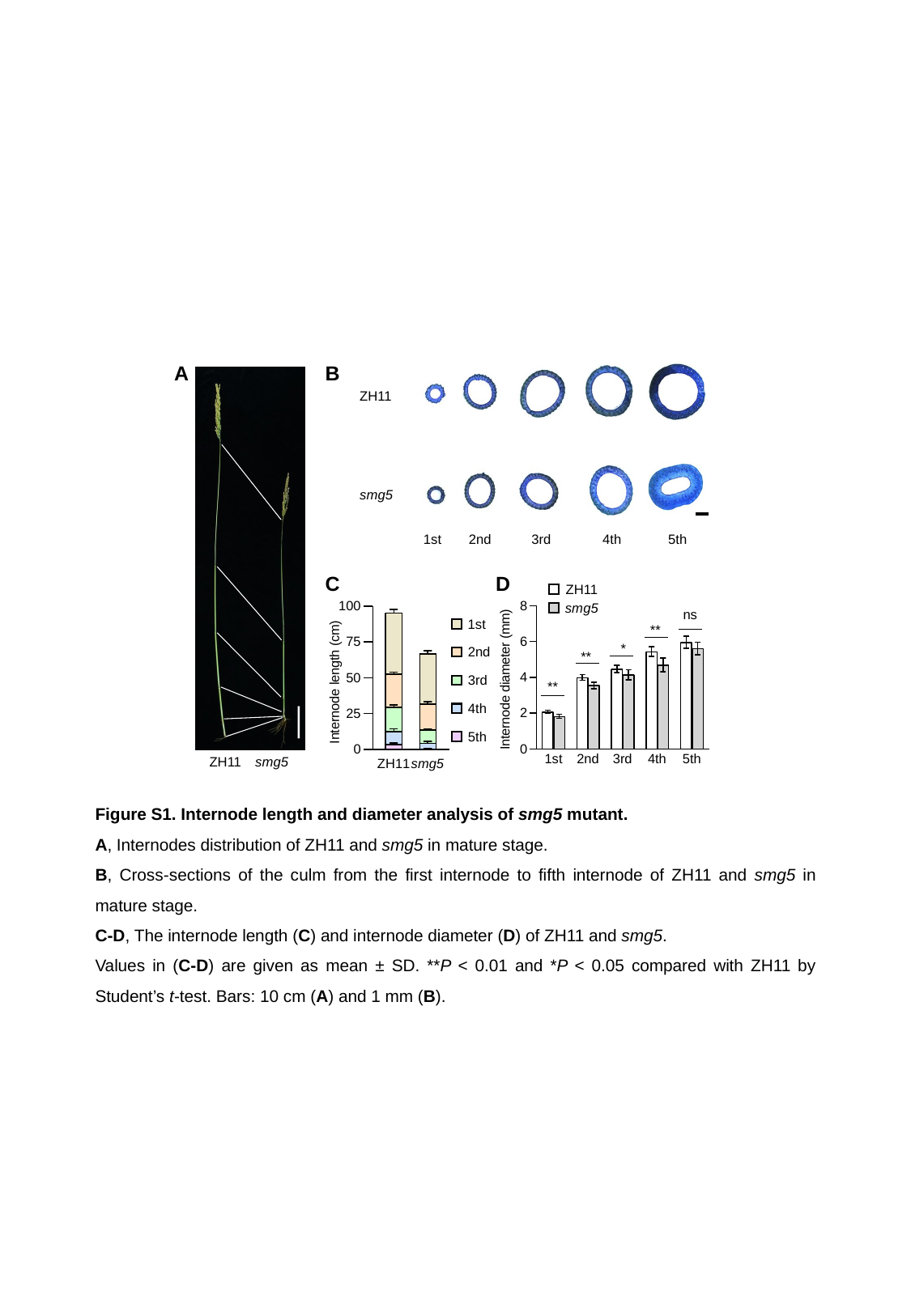

A
B
ZH11
smg5
1st
2nd
3rd
4th
5th
smg5
C
D
ZH11
smg5
ns
**
*
**
**
1st
2nd
3rd
4th
5th
ZH11
smg5
Figure S1. Internode length and diameter analysis of smg5 mutant.
A, Internodes distribution of ZH11 and smg5 in mature stage.
B, Cross-sections of the culm from the first internode to fifth internode of ZH11 and smg5 in mature stage.
C-D, The internode length (C) and internode diameter (D) of ZH11 and smg5.
Values in (C-D) are given as mean ± SD. **P < 0.01 and *P < 0.05 compared with ZH11 by Student’s t-test. Bars: 10 cm (A) and 1 mm (B).

## Slide 2
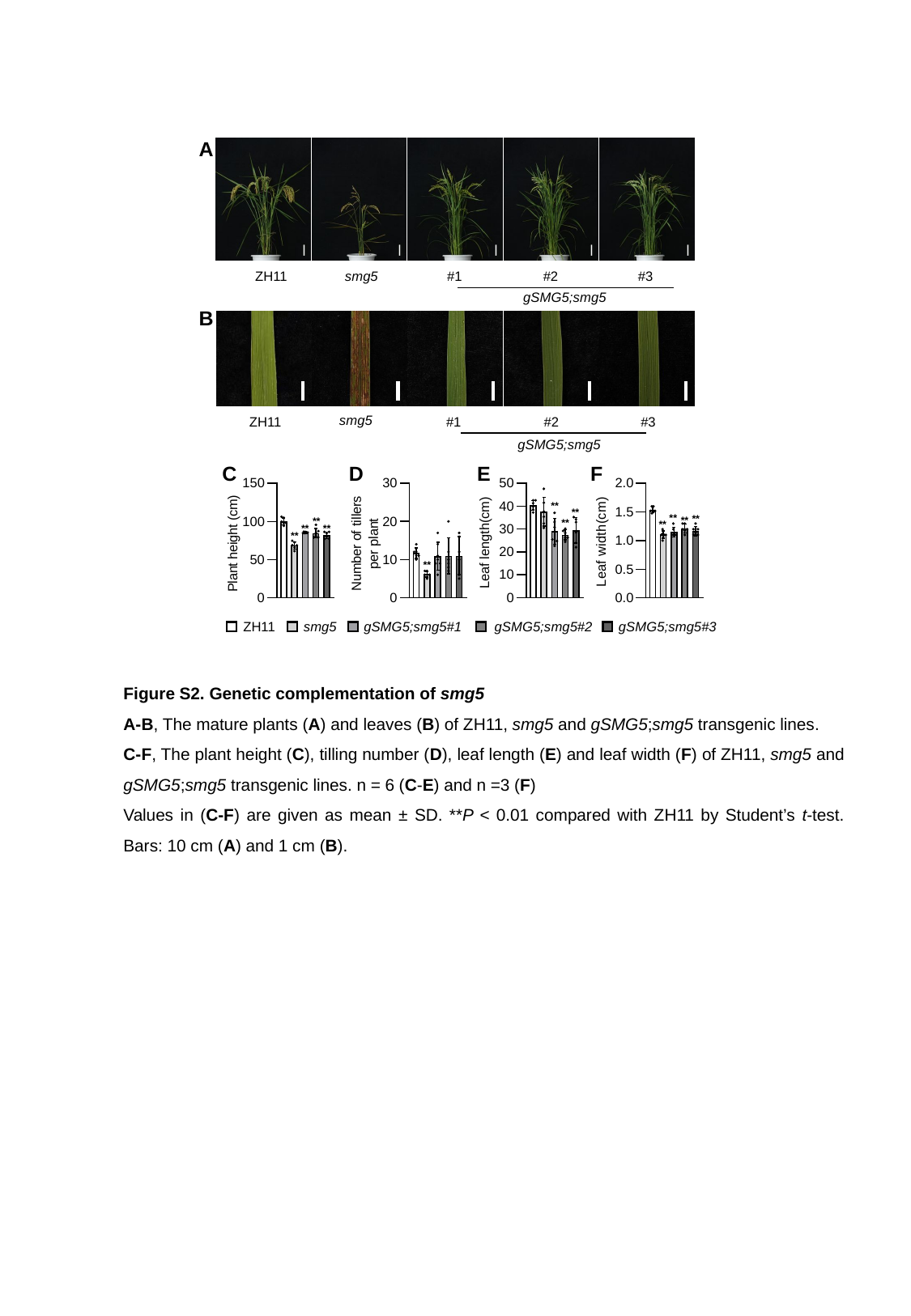

A
ZH11
smg5
#1
#2
#3
gSMG5;smg5
B
smg5
ZH11
#1
#2
#3
gSMG5;smg5
C
D
E
F
**
**
**
**
**
**
**
**
**
**
**
**
ZH11
smg5
gSMG5;smg5#1
gSMG5;smg5#2
gSMG5;smg5#3
Figure S2. Genetic complementation of smg5
A-B, The mature plants (A) and leaves (B) of ZH11, smg5 and gSMG5;smg5 transgenic lines.
C-F, The plant height (C), tilling number (D), leaf length (E) and leaf width (F) of ZH11, smg5 and gSMG5;smg5 transgenic lines. n = 6 (C-E) and n =3 (F)
Values in (C-F) are given as mean ± SD. **P < 0.01 compared with ZH11 by Student’s t-test. Bars: 10 cm (A) and 1 cm (B).

## Slide 3
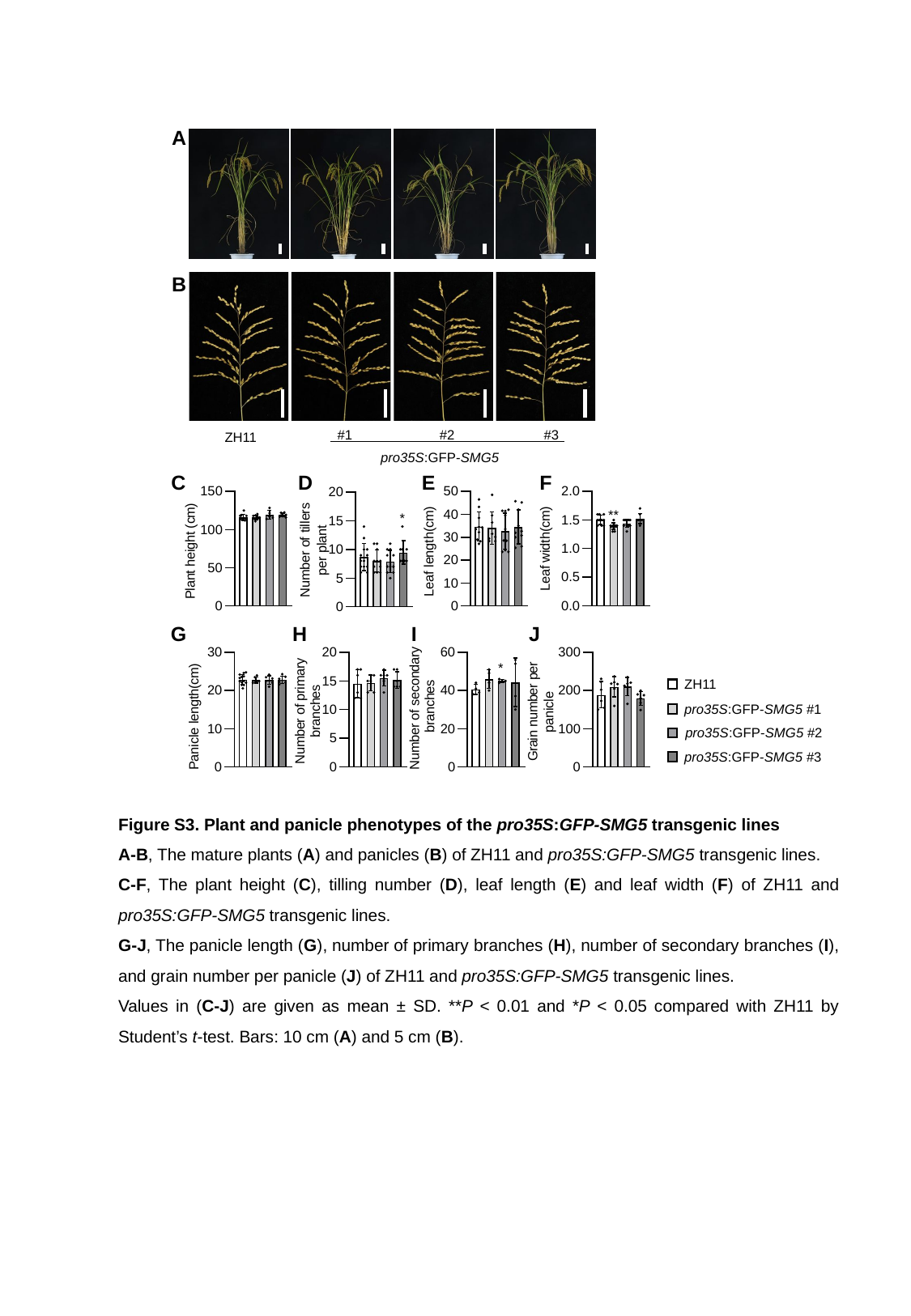

A
B
#1
#2
#3
ZH11
pro35S:GFP‑SMG5
C
D
E
F
**
*
G
H
I
J
*
ZH11
pro35S:GFP‑SMG5 #1
pro35S:GFP‑SMG5 #2
pro35S:GFP‑SMG5 #3
Figure S3. Plant and panicle phenotypes of the pro35S:GFP-SMG5 transgenic lines
A-B, The mature plants (A) and panicles (B) of ZH11 and pro35S:GFP-SMG5 transgenic lines.
C-F, The plant height (C), tilling number (D), leaf length (E) and leaf width (F) of ZH11 and pro35S:GFP-SMG5 transgenic lines.
G-J, The panicle length (G), number of primary branches (H), number of secondary branches (I), and grain number per panicle (J) of ZH11 and pro35S:GFP-SMG5 transgenic lines.
Values in (C-J) are given as mean ± SD. **P < 0.01 and *P < 0.05 compared with ZH11 by Student’s t-test. Bars: 10 cm (A) and 5 cm (B).

## Slide 4
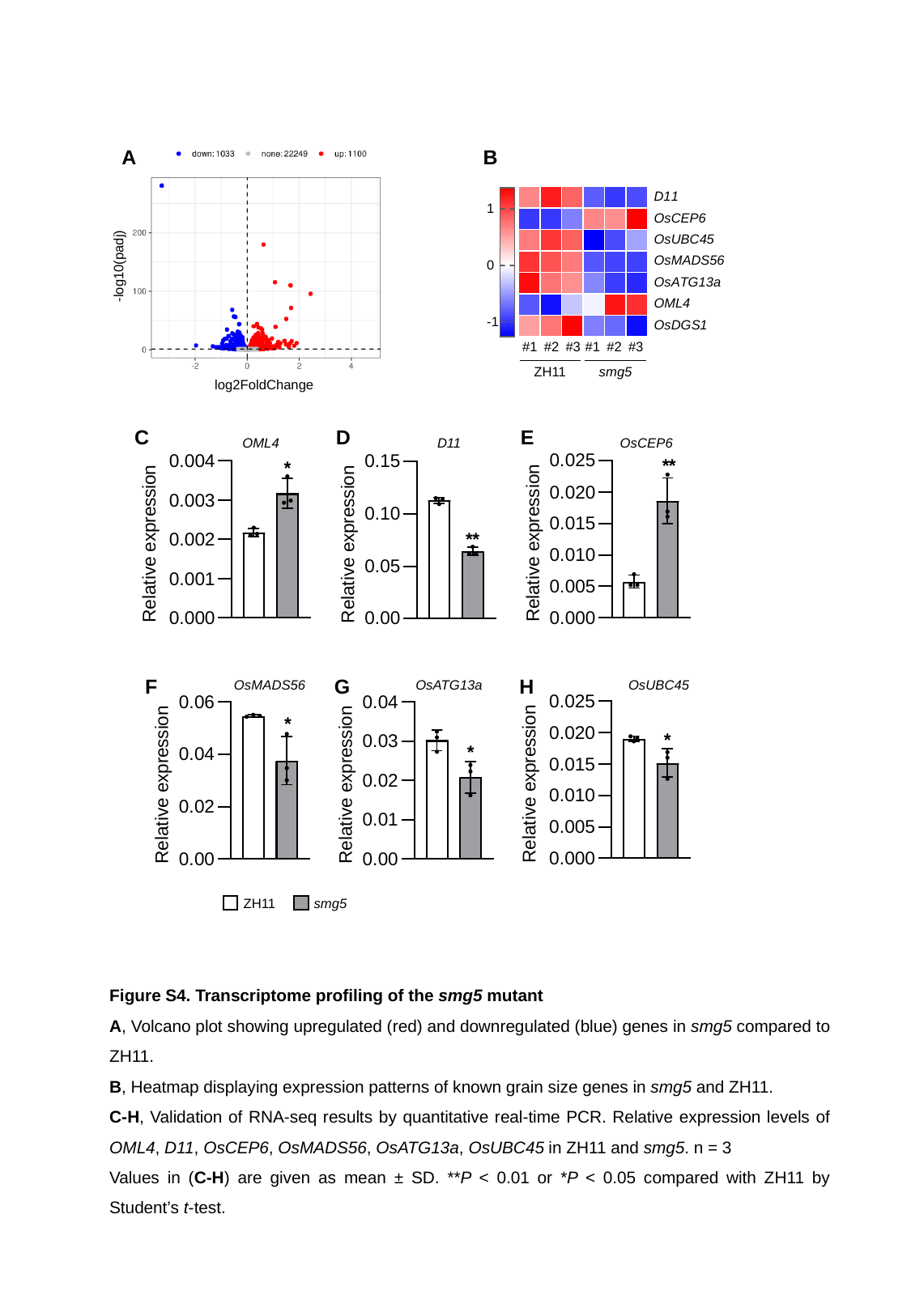

A
B
-log10(padj)
log2FoldChange
#1
#2
#3
#1
#2
#3
ZH11
smg5
C
D
E
OML4
D11
OsCEP6
OsMADS56
OsATG13a
OsUBC45
F
G
H
ZH11
smg5
Figure S4. Transcriptome profiling of the smg5 mutant
A, Volcano plot showing upregulated (red) and downregulated (blue) genes in smg5 compared to ZH11.
B, Heatmap displaying expression patterns of known grain size genes in smg5 and ZH11.
C-H, Validation of RNA-seq results by quantitative real-time PCR. Relative expression levels of OML4, D11, OsCEP6, OsMADS56, OsATG13a, OsUBC45 in ZH11 and smg5. n = 3
Values in (C-H) are given as mean ± SD. **P < 0.01 or *P < 0.05 compared with ZH11 by Student’s t-test.

## Slide 5
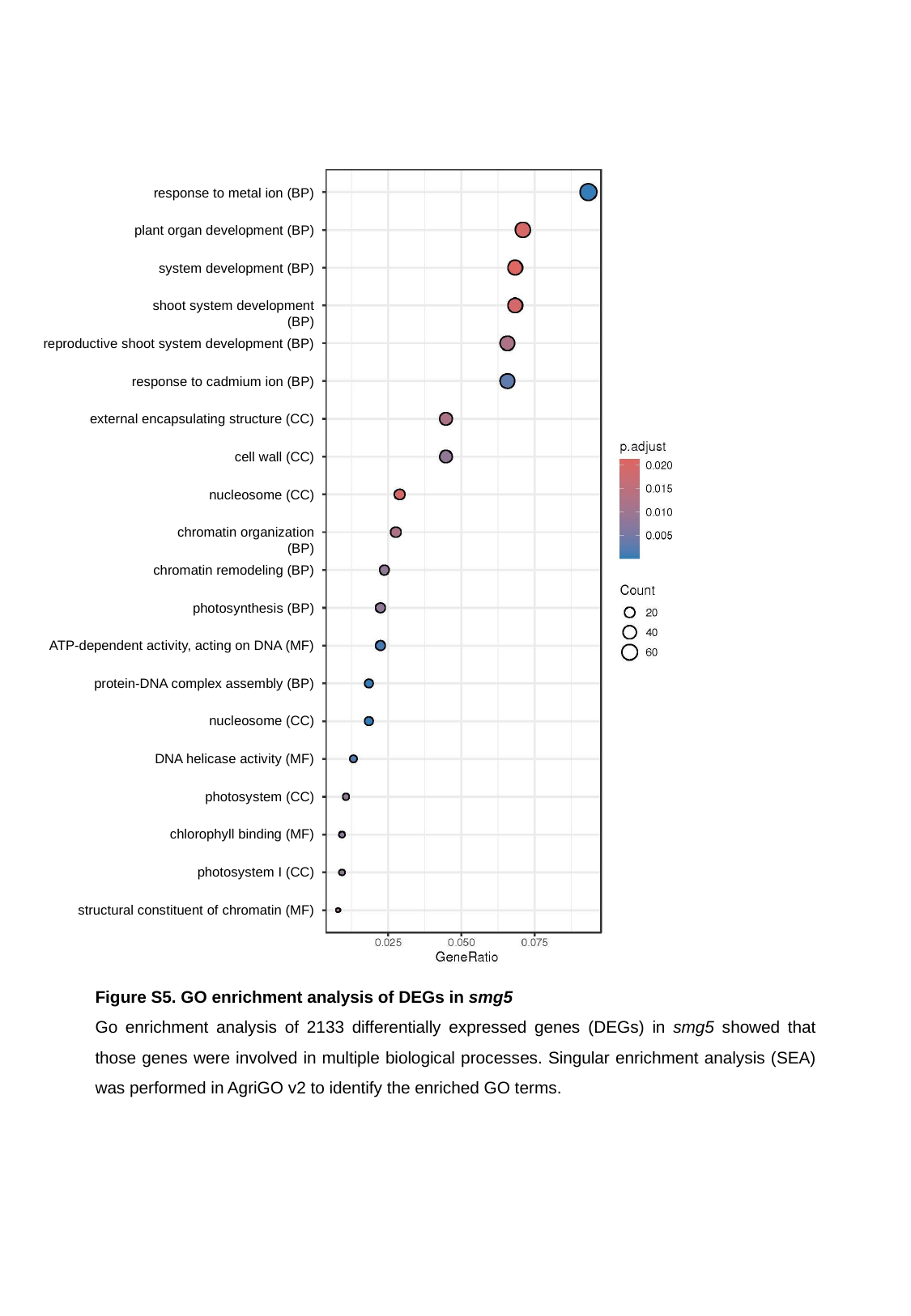

response to metal ion (BP)
plant organ development (BP)
system development (BP)
shoot system development (BP)
reproductive shoot system development (BP)
response to cadmium ion (BP)
external encapsulating structure (CC)
cell wall (CC)
nucleosome (CC)
chromatin organization (BP)
chromatin remodeling (BP)
photosynthesis (BP)
ATP-dependent activity, acting on DNA (MF)
protein-DNA complex assembly (BP)
nucleosome (CC)
DNA helicase activity (MF)
photosystem (CC)
chlorophyll binding (MF)
photosystem I (CC)
structural constituent of chromatin (MF)
Figure S5. GO enrichment analysis of DEGs in smg5
Go enrichment analysis of 2133 differentially expressed genes (DEGs) in smg5 showed that those genes were involved in multiple biological processes. Singular enrichment analysis (SEA) was performed in AgriGO v2 to identify the enriched GO terms.

## Slide 6
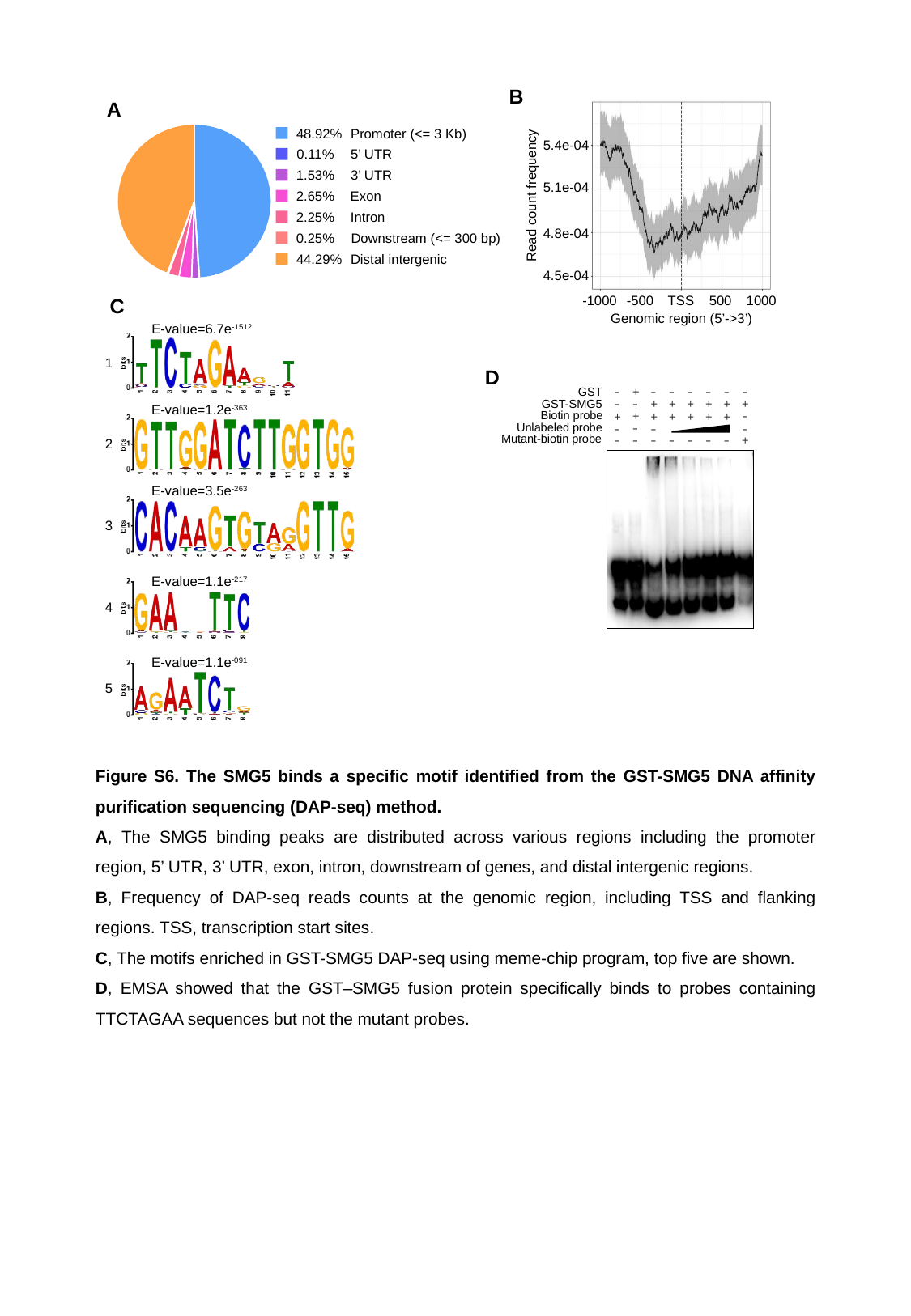

B
5.4e-04
5.1e-04
Read count frequency
4.8e-04
4.5e-04
-1000
-500
TSS
500
1000
Genomic region (5’->3’)
A
48.92%
Promoter (<= 3 Kb)
0.11%
5’ UTR
1.53%
3’ UTR
2.65%
Exon
2.25%
Intron
0.25%
Downstream (<= 300 bp)
44.29%
Distal intergenic
C
E-value=6.7e-1512
1
E-value=1.2e-363
2
E-value=3.5e-263
3
E-value=1.1e-217
4
E-value=1.1e-091
5
D
–
–
+
–
–
+
–
+
–
–
–
+
+
–
–
–
+
+
–
–
+
+
–
–
+
+
–
–
+
+
–
–
+
–
–
+
GST
GST-SMG5
Biotin probe
Unlabeled probe
Mutant-biotin probe
Figure S6. The SMG5 binds a specific motif identified from the GST-SMG5 DNA affinity purification sequencing (DAP-seq) method.
A, The SMG5 binding peaks are distributed across various regions including the promoter region, 5’ UTR, 3’ UTR, exon, intron, downstream of genes, and distal intergenic regions.
B, Frequency of DAP-seq reads counts at the genomic region, including TSS and flanking regions. TSS, transcription start sites.
C, The motifs enriched in GST-SMG5 DAP-seq using meme-chip program, top five are shown.
D, EMSA showed that the GST–SMG5 fusion protein specifically binds to probes containing TTCTAGAA sequences but not the mutant probes.
